# Combined Dietary Anthocyanins, Flavonols, and Stilbenoids Alleviate Inflammatory Bowel Disease Symptoms in Mice

**DOI:** 10.3389/fnut.2017.00075

**Published:** 2018-01-24

**Authors:** Aurelia Scarano, Eugenio Butelli, Stefania De Santis, Elisabetta Cavalcanti, Lionel Hill, Maria De Angelis, Giovanna Giovinazzo, Marcello Chieppa, Cathie Martin, Angelo Santino

**Affiliations:** ^1^ISPA-CNR, Institute of Science of Food Production, C.N.R. Unit of Lecce, Lecce, Italy; ^2^John Innes Centre, Colney Research Park, Norwich, United Kingdom; ^3^National Institute of Gastroenterology “S. de Bellis”, Institute of Research, Castellana Grotte, Bari, Italy; ^4^Department of Soil, Plant and Food Sciences, University of Bari, Bari, Italy

**Keywords:** polyphenols, tomato, metabolic engineering, microbiota, inflammatory bowel disease

## Abstract

Dietary polyphenols are associated with a wide range of health benefits, protecting against chronic diseases and promoting healthy aging. Dietary polyphenols offer a complementary approach to the treatment of inflammatory bowel diseases (IBDs), a group of common chronic intestinal inflammation syndromes for which there is no cure. Tomato is widely consumed but its content of polyphenols is low. We developed a tomato variety, Bronze, enriched in three distinct classes of polyphenols: flavonols, anthocyanins, and stilbenoids. Using Bronze tomatoes as a dietary supplement as well as Indigo (high anthocyanins and flavonols), ResTom (high stilbenoids) and wild-type tomatoes, we examined the effects of the different polyphenols on the host gut microbiota, inflammatory responses, and the symptoms of chronic IBD, in a mouse model. Bronze tomatoes significantly impacted the symptoms of IBD. A similar result was observed using diets supplemented with red grape skin containing flavonols, anthocyanins, and stilbenoids, suggesting that effective protection is provided by different classes of polyphenols acting synergistically.

## Introduction

Polyphenols represent a large family of plant secondary metabolites. They include flavonols, anthocyanins, catechins, tannins, and stilbenoids, and are present in many types of fruit, vegetables, and beverages, which are consumed as a regular part of the human diet. Interest in dietary polyphenols and in the production and consumption of polyphenol-rich foods is increasing because of their health-promoting properties (1).

Dietary polyphenols can regulate intracellular signaling pathways to counteract oxidative damage, which is implicated in the onset of degenerative diseases and chronic inflammation. Polyphenols can also modulate the intestinal immune response and the production of molecular mediators of inflammation (2–6).

Several studies indicate an association between dietary polyphenols and a reduction in the severity of symptoms of inflammatory bowel disease (IBD) (7, 8). IBD includes Crohn’s disease and ulcerative colitis, which are immune-mediated chronic conditions of the gastrointestinal tract, dependent on complex interactions between factors determining genetic susceptibility, environmental factors and gut microbiota (9). IBD is estimated to affect 2.2 million Europeans and 1.5 million Americans (9, 10). Although lower than in industrialized countries, the incidence and prevalence of IBD are also rising in developing countries and have been linked to the westernization of diet and lifestyle (11). There is no effective cure for IBD but immune-modulator and anti-inflammatory drugs (such as aminosalicylates, azathioprine, and corticosteroids) and monoclonal antibodies targeted toward cytokines and adhesion molecules (such as infliximab, adalimumab, vedolizumab) are often used in treatment (12). Since diet can influence the pathogenesis of IBD, nutritional intervention is now considered an important adjunct strategy for more effective treatment of IBD.

The effects of specific polyphenols on gut inflammation have been investigated with both *in vitro* and *in vivo* models using purified compounds (2, 8). This approach has obvious limitations, ranging from the prohibitive cost of supplying large amounts of compounds in purified form to the fact that the food matrix and the interaction with other nutritional components can have profound effects on the bioavailability and bioactivity of dietary polyphenols (13).

Tomatoes accumulate low levels of polyphenols mostly in the skin. High levels of different polyphenols, including compounds completely absent in cultivated varieties, have been engineered in tomatoes by expressing biosynthetic and/or regulatory genes encoding transcription factors (14–18). MYB12, a transcription factor from *Arabidopsis thaliana*, has been used as an effective tool in metabolic engineering to enhance the biosynthesis of polyphenols in tomatoes (17, 18). The co-expression of *AtMYB12* either with other regulatory genes (*Delila* and *Rosea1* from *Antirrhinum majus*), or with structural genes committed to different branches of the phenylpropanoid pathway [stilbene synthase (*StSy*) from *Vitis vinifera* or isoflavone synthase from *Lotus japonicus*] has been used to generate tomato lines enriched in specific classes of polyphenols (18).

## Materials and Methods

### Ethics Statement

Our investigations have been conducted in accordance with national and international guidelines and were approved by the authors’ institutional review board (Organism for Animal Wellbeing––OPBA). Female mice between 6- and 8-week-old were purchased from Jackson Laboratories: Wild-type C57BL/6 (Stock no: 000664; weight: approximately 20 g). All animal experiments were carried out in accordance with Directive 86/609 EEC enforced by Italian D.L. n. 116 1992, and approved by the Committee on the Ethics of Animal Experiments of Ministero della Salute––Direzione Generale Sanità Animale (2012/00000923 A00: Eo_GINRC) and the official RBM veterinarian. Animals were sacrificed if found in a severe clinical condition in order to avoid undue suffering.

### Generation of Tomato Lines and Diets

The Bronze tomato line (*E8:MYB12, E8:Del/Ros, 35S:StSy*) was developed by crossing two lines generated as described by Zhang et al. (18): Indigo (*E8:MYB12, E8:Del/Ros*) and ResTom (*E8:MYB12, 35S:StSy*) were used as the male and as the female parent, respectively. The resulting plants were screened by PCR for the presence of the four transgenes. From seeds of selected plants, screened seedlings were raised under greenhouse conditions. Whole mature fruits were freeze dried, ground into a fine powder, and incorporated in to the rodent diets. The standard rodent pellet (purchased from Mucedola) was soaked in distilled water, the tomato freeze-dried powder was added, and the mixture was manually homogenized. The final pellet was dried in a ventilated cabinet for 48 h and stored in vacuum packing until the use.

### Identification and Characterization of Polyphenols

Whole tomato fruits were frozen in liquid nitrogen, freeze dried and finely ground. Moreover, 200 mg of powder were extracted twice with 5-mL 80% methanol at 4°C, overnight. Extracts were centrifuged at 5,000× *g* for 20 min at 4°C and supernatants were collected. Supernatants were filtered through a 0.22-μ filter and then stored at −20°C until use.

Extracts were run on a Shimadzu Nexera LC system attached to an IT ToF mass spectrometer. Separation was on a 100 mm × 2.1 mm 2.6-μ Kinetex XB-C18 column (Phenomenex), using the following gradient of acetonitrile (ACN) versus 0.1% formic acid, run at 0.5 mL·min^−1^ and 40°C: 0 min, 2% ACN; 0.5 min, 2% ACN; 3 min, 10% ACN; 13 min, 30% ACN; 18 min, 90% ACN; 18.8 min, 90% ACN; 19 min, 2% ACN; 23.1 min, 2% ACN. Detection was by UV/visible absorbance, collecting spectra from 200 to 600 nm, from which extracted ion chromatograms could be taken at appropriate wavelengths for each analyte. The instrument also collected positive electrospray MS, with spectra from *m*/*z* 200 to 2,000 and MS2 spectra of the most abundant precursors, collected at an isolation width of *m*/*z* 3.0, and fragmented at 50% collision energy and 50% gas. Spray chamber conditions were 250°C curved desorption line temperature, 1.5 L·min^−1^ nebulizing gas, and 300°C heat block. The instrument was calibrated with sodium trifluoroacetate before use according to the manufacturer’s instructions.

For anthocyanin quantification, powder of fresh fruits was extracted with acidified (0.5% HCl v/v) 80% methanol, as previously described (16).

### Murine Models

Sex- and weight-matched mice were divided into five groups (five mice each). Mice, pellet consumption, and drinking water were monitored on a daily basis. Each group of mice received a different diet. Freeze-dried tomato or grape powder was supplemented by addition to a standard rodent diet at 1% (tomato based-diets; see also Supplementary Methods in Supplementary Material). Groups of mice were fed with the different tomato-supplemented diets for 2 weeks. Chronic colitis was induced by administration of 1% DSS in drinking water, starting from day 14. Body weight (BW), stool consistency, and rectal bleeding were recorded. Mice were sacrificed at day 29, and colon and mesenteric lymph node (MLN) tissues were explanted to evaluate clinical severity of colitis. Colon length was measured as an indicator of colonic inflammation. The colon/BW index was calculated as the ratio of the colon wet weight and the total BW of each mouse. BW, occult and rectal bleeding, and stool consistency were monitored daily after DSS administration. Disease activity index (DAI) was determined by scoring changes in BW, occult blood, and gross bleeding.

### Generation and Culture of Dendritic Cells (DCs)

Dendritic cells were harvested from murine bone marrow. Briefly, bone marrows from the tibiae and femurs of 6- to 8-week-old male C57Bl/6 mice were flushed with RPMI and depleted of red blood cells with ACK cell lysing buffer (GIBCO). The cells were plated in 6-well culture plates (1Å–10^6^ cells/mL; 3 mL/well) in RPMI supplemented with 10% heat-inactivated FBS, 100-U·mL^−1^ penicillin, 100-mg·mL^−1^ streptomycin, 25-µg·mL^−1^ rmGM-CSF, and 25-µg·mL^−1^ rmIL-4 at 37°C in a humidified 5% CO2 atmosphere. On day 3, bone marrow DCs (BMDCs) were harvested and plated at 1 × 10^6^ mL^−1^ on 24-well culture plates. On day 7, BMDCs were administered with tomato methanol extracts (0.1-g lyophilized powder·mL^−1^) at a 1:25 final dilution. Lipopolysaccharide (LPS) was administered [1 µg·mL^−1^] at day 8 for 24 h. DC viability was assessed by cytofluorimetric analysis (Supplementary Methods in Supplementary Material).

### Enzyme-Linked Immunosorbent Assay (ELISA)

Bone marrow dendritic cells were analyzed for IL-6 and TNFα proteins in triplicate, using an ELISA kit, as described by the manufacturer (R&D Systems, Minneapolis, MN, USA).

### 454 Pyrosequencing of 16S rRNAs

Stools were collected from each mouse separately and used for 16S rRNA sequencing for microbiome evaluation. The following 27F and 533R universal primers were used for PCR amplification of the V1–V3 hypervariable regions of the 16S rRNA gene. These two primers have been recommended by the NIH RoadMap Human Microbiome Project. Primer 533R included a unique sequence tag to barcode the samples, enabling 100 specimens to be run as one batch. The primers were as follows:

27F-5′-*CCATCTCATCCCTGCGTGTCTCCGACTCAGTCAGA***GTTTGATCCTGGCTCAG**-3′,
533R-5′-*CCTATCCCCTGTGTGCCTTGGCAGTCTCAGNN*NNNNNNNNNNCAT**TACCGCGGCTGCTGGCA**-3′,

where the italicized and underlined sequences were the 454 Life Sciences^®^ Titanium sequencing primers B and A in 27F and 533R, respectively, and the bold font denotes the universal 16S rRNA primers 27F and 533R. The 12-bp barcode within primer 533R is denoted by 12 N’s. 16S rRNA genes were amplified in 96-well microtiter plates. Negative controls without a template were run for each primer pair. The presence of amplicons was confirmed and quantified using the QIAxcel System (Qiagen) and equimolar amounts (~100 ng) of the PCR amplicons mixed in a single tube. Amplification primers and reaction buffer were removed by processing the amplicons mixture with the AMPure Kit (Agencourt). The purified amplicon mixtures were sequenced by 454 FLX Titanium pyrosequencing at the Genomics4life (Baronissi, Salerno, IT) core facility.

Quality control (QC) and taxonomic assignments were undertaken according to the QIIME and the Ribosomal Database Project Bayesian classifier in combination with a set of custom designed informatics pipelines implemented by Genomix4life for analyses of microbial communities. Alpha-diversity indexes were evaluated using the number of OTUs, Chao1 species richness, and the Shannon index.

### Statistical Analysis

All data were expressed as the means ± SEM. Statistical analysis of the relative abundances of microbial genera was based on Duncan’s multiple-range test, with a significance level of 0.05. Finally, unless specifically described, other data and group differences were analyzed and compared by paired or unpaired, two-tailed Student’s *t*-tests.

## Results

### Generation of Tomato Fruit Enriched in Different Types of Polyphenols

**Figure 1:**
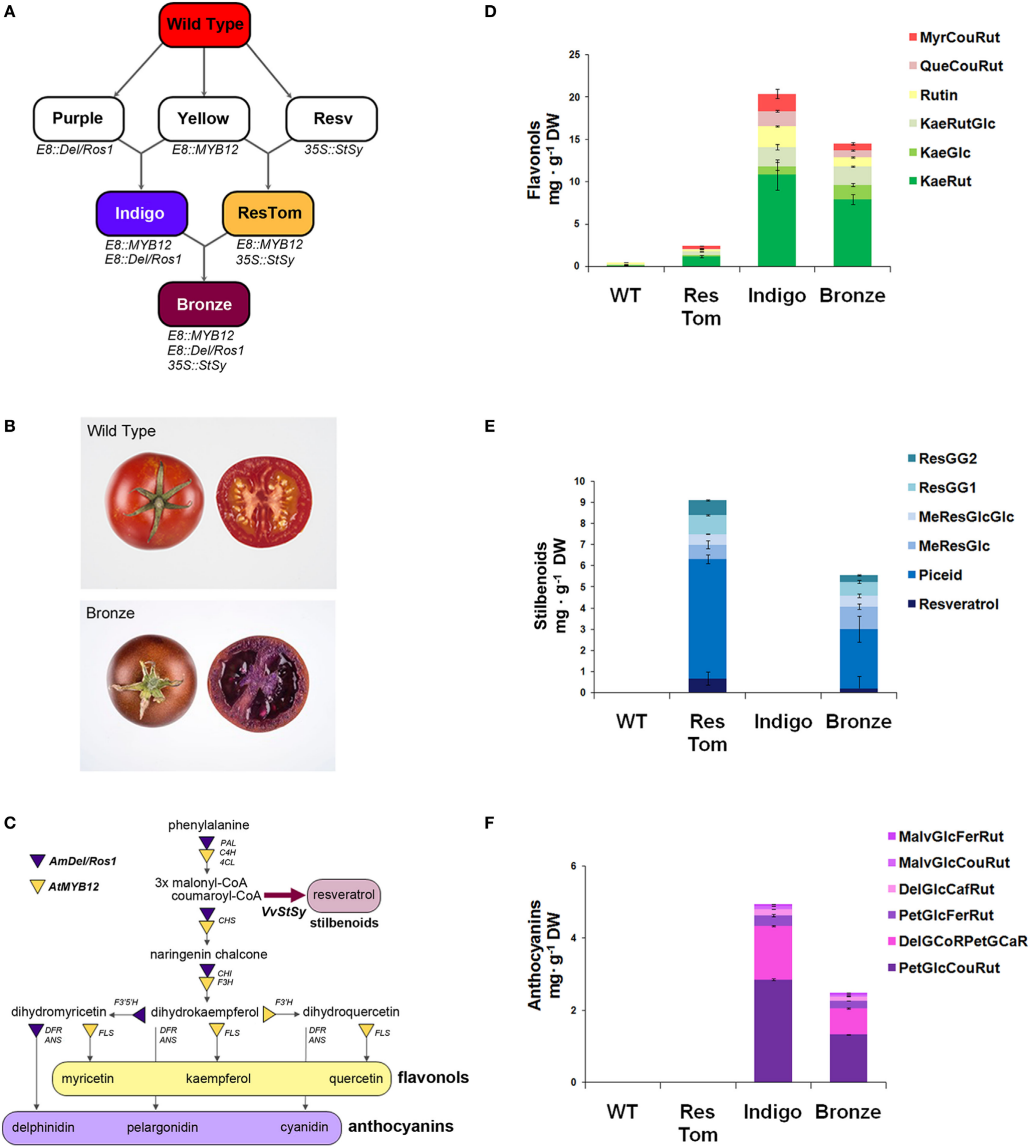
Metabolically engineered tomato lines expressing a combination of three regulatory genes with a fourth structural gene, involved in different branches of the phenylpropanoid pathway. **(A)** Breeding strategy adopted to develop Indigo, ResTom, and Bronze tomatoes. **(B)** Whole and cross-section of wild-type and Bronze tomato fruit. **(C)** Schematic representation of the general phenylpropanoid biosynthetic pathway showing the different branches activated in Bronze fruit by the introduction of the *AtMYB12, AmDel, AmRos1*, and *VvStSy*. PAL, phenylalanine ammonia lyase; 4CL, 4-coumarate-coenzyme A ligase; C4H, cinnamate-4-hydroxylase; CHS, chalcone synthase; CHI, chalcone isomerase; F3H, flavanone-3-hydroxylase; F3’H, flavonoid-3′-hydroxylase; F3′5 ′H, flavonoid-3′5 ′-hydroxylase; FLS, flavonol synthase; DFR, dihydroflavonol reductase; ANS, anthocyanidin synthase. **(D–F)** Quantification by LC/MS analysis of flavonols **(D)**, stilbenoids **(E)**, and anthocyanins **(F)**, in Bronze, compared with wild-type, Indigo and ResTom tomato fruit. Error bars show SEM (*n* = 3). Chromatograms and details of compound identification by LC/MS are provided in Figures S1–S3 and Tables S1–S3 in Supplementary Material.

With the aim of assessing the potential health benefits of different classes of polyphenols (flavonols, stilbenoids, anthocyanins), within the same food matrix on an IBD mouse model, we developed an additional tomato line by sequential crossing of the three metabolically engineered varieties previously described (15–18). As illustrated in Figure 1A, the resulting line, named Bronze because of the metallic brown color of the ripe fruit skin (Figure 1B), expressed the regulatory genes *Delila* and *Rosea1* from *A. majus* that induce anthocyanin biosynthesis, *MYB12* from *A. thaliana*, regulating flavonol biosynthesis and the biosynthetic gene *StSy* from *V. vinifera* that is essential for the production of resveratrol and related stilbenoids (Figure 1C). The regulatory genes were expressed under the control of the fruit-specific promoter E8 from tomato (*E8:Del/Ros1*and *E8:MYB12*), while the expression of *StSy* was driven by the constitutive promoter from cauliflower mosaic virus CaMV35S (*35S:VvStSy*). Two parental varieties were used to generate Bronze: Indigo (*E8:MYB12, E8:Del/Ros1*) and ResTom (*E8:MYB12, 35S:VvStSy*) as described in Zhang et al. (18) (Figure 1A).

Polyphenols in the fruit of Bronze in the fruit of parental lines Indigo and ResTom and in wild-type tomatoes were analyzed by LC/MS (Figures 1D–F; Figures S1–S3 in Supplementary Material). The Bronze line accumulated substantial levels of flavonols, stilbenoids, and anthocyanins in the fruit. Compared with parental lines where the highest amounts were recorded, the Bronze fruit showed the presence of about 30–50% lower levels of flavonols and anthocyanins than the Indigo line and 30–50% lower levels of stilbenoids than the ResTom line (Figures 1D–F). The presence of substantial levels of different polyphenols in the Bronze fruit paralleled an increase in antioxidant capacity of methanol extracts of fruit, assessed by Trolox equivalent antioxidant capacity (TEAC) and oxygen radical absorbance capacity (ORAC) assays (Figure S4 in Supplementary Material). In contrast to these changes in hydrophilic antioxidant capacity, no significant differences were found in the antioxidant capacity of the lipophilic ethyl acetate fractions nor in the total carotenoid content of fruit from the different tomato lines (Figures S4 and S5 in Supplementary Material).

### Diets Supplemented with Polyphenol-Enriched Tomatoes Cause Changes in the Composition of Gut Microbiota in Healthy Mice

**Figure 2:**
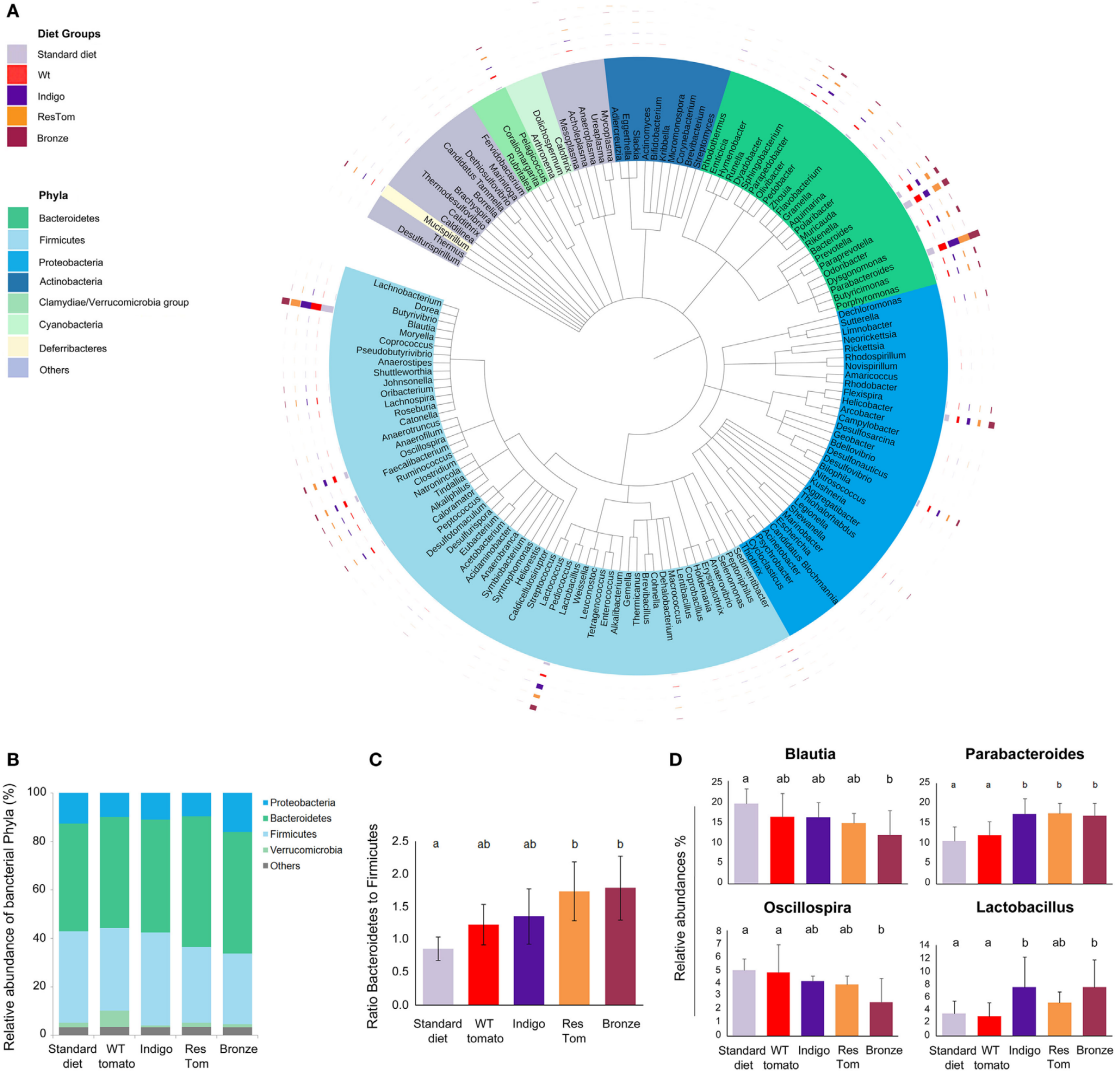
Composition of the intestinal microbiota of mice fed diets enriched with different tomato fruit. Sex-matched mice (*n* = 5) were divided into five groups, based on the diet (standard diet and diets enriched with 1% of wild-type, Indigo, ResTom, and Bronze lyophilized tomato fruit). After 2 weeks diet, fecal samples were collected for the microbiota meta-analysis. **(A)** Circular representation of the phylogenetic tree showing the major 150 genera of the intestinal microbiota. The inner bands indicate the genera colored by phylum. The outer circles show multibar charts indicating the relative abundances of genera in different diet groups. **(B)** Relative abundances of the phyla found in the microbiome of mice in different diet groups. **(C)** Bacteroidetes/Firmicutes ratio in the microbiome of mice fed with different diets. **(D)** Relative abundances of the genera of the microbiome showing significant differences among diet groups. The letters above the histograms in panels **(C)** and **(D)** indicate the significant differences between two different diet groups assessed by Duncan’s multiple-range test.

We investigated any impact of the different tomato-supplemented diets on probiotic groups, whose growth is relevant to intestinal health (4, 8, 19, 20). We first verified that lyophilized fruit of different tomato lines did not impair the *in vitro* growth rates of a number of probiotic *Lactobacillus* and *Bifidobacterium* strains. Compared with controls, all the media supplemented with lyophilized tomato powders increased the rate of bacterial cell growth (Figure S6 in Supplementary Material). We then evaluated the effects of tomato fruits from the different lines as dietary supplements on the composition of the gut microbiota. We developed different custom diets supplemented with 1% lyophilized fruit from the different tomato lines (wild type, Indigo, ResTom, and Bronze) and administered them to age- and sex-matched C57Bl/6 mice (five mice *per* diet, *n* = 5), for 2 weeks. Mice were monitored three times *per* week for weight gain, general health, and food and water consumption. Stools from each mouse were collected at day 13 and 14 and used to extract total DNA from which a complete metagenomic analysis of the microbiota was carried out. The 454 pyrosequencing of 16S rRNA genes was performed on fecal samples, to quantify the abundance of intestinal microbial genera and verify bacterial ecology in terms of overall biodiversity and co-occurrence of genera. The microbial diversity and richness in all samples were comparable as assessed using the Shannon and Chao1 indices (21) and did not show significant differences (*P* > 0.05) in response to different diets (Table S4 in Supplementary Material). However, 16S meta-analysis revealed a consistent shift in the relative abundance of specific bacterial phyla or genera (Figure 2; Figure S5 in Supplementary Material). In particular, mice fed with diets enriched with Bronze and ResTom tomato fruit were characterized by a significant increase in the phylum *Bacteroidetes* and a decrease in *Firmicutes*, resulting in a significant increase (*P* < 0.05) in the ratio of *Bacteroidetes* to *Firmicutes* which doubled from 0.86 in mice fed the standard diet to 1.74–1.79 in mice fed diets enriched with 1% ResTom and 1% Bronze tomato fruit, respectively (Figure 2C). These data suggested that dietary stilbenoids promoted growth of *Bacteroidetes* over *Firmicutes* in mice.

A closer examination of different bacterial genera revealed that the population of *Parabacteroides* was significantly increased on all the diets enriched in polyphenols (Indigo, 17.31%; ResTom, 17.53%; Bronze 17.01%) compared with wild-type tomato-supplemented and standard diets (12.15 and 10.77%, respectively). Similarly, *Lactobacilli* were significantly higher in animals fed with the Indigo or Bronze tomato-supplemented diets (7.51% for both) compared with the wild-type tomato-supplemented diets (3.01%; Figure 2D), suggesting that polyphenols promote the growth of *Lactobacilli* amongst the genera of the murine microbiota. A significant decrease (*P* < 0.05) in *Blautia* and a decrease in *Oscillospira* genera were found in all the groups fed with tomato-enriched diets compared with the standard diet (where Blautia represented 19.5% and Oscillospira represented 5% of the microbiota, respectively), particularly for the Bronze diet (where Blautia represented 11.8% and Oscillospira 2.6%, respectively) (Figure 2).

To analyze the effect of diet on the interactions between polyphenols and components of the microbial communities, we performed two different correlation analyses, which showed significant changes in the co-occurrence of different genera in response to different diets (Table S6 and Figure S8 in Supplementary Material).

Taken together, these results indicated that the supplementation of different types of polyphenols in the diet can “re-shape” the gut microbiota in terms of composition, favoring the growth of specific genera and remodeling microbial associations and communities.

Besides their influence on the gut microbiota, polyphenols are able to induce changes in epithelial and immune cells of the host’s intestine (3–5, 22, 23). Diets enriched in these bioactive compounds may determine changes in gene expression profiles of the host’s gastrointestinal tract. To assess whether this occurred in response to the different tomato-supplemented diets, we analyzed gene expression patterns using mRNA extracted from the colons of the mice after the different dietary interventions (Figure S8 in Supplementary Material). As expected, we did not observe a marked upregulation of genes involved in the inflammatory pathway, but we found unique changes in the gene expression profile following the nutritional intervention with Bronze tomatoes. These changes included the downregulation of SOCS3, a positive regulator of cytokine signaling (24), Muc1, whose overexpression is associated with IBD (25) and S100a8, a component of the calprotectin complex and a clinical marker for IBD (26) (Figure S8 in Supplementary Material).

### A Bronze Tomato-Supplemented Diet Ameliorated the Symptoms of DSS-Induced Colitis in Mice

**Figure 3:**
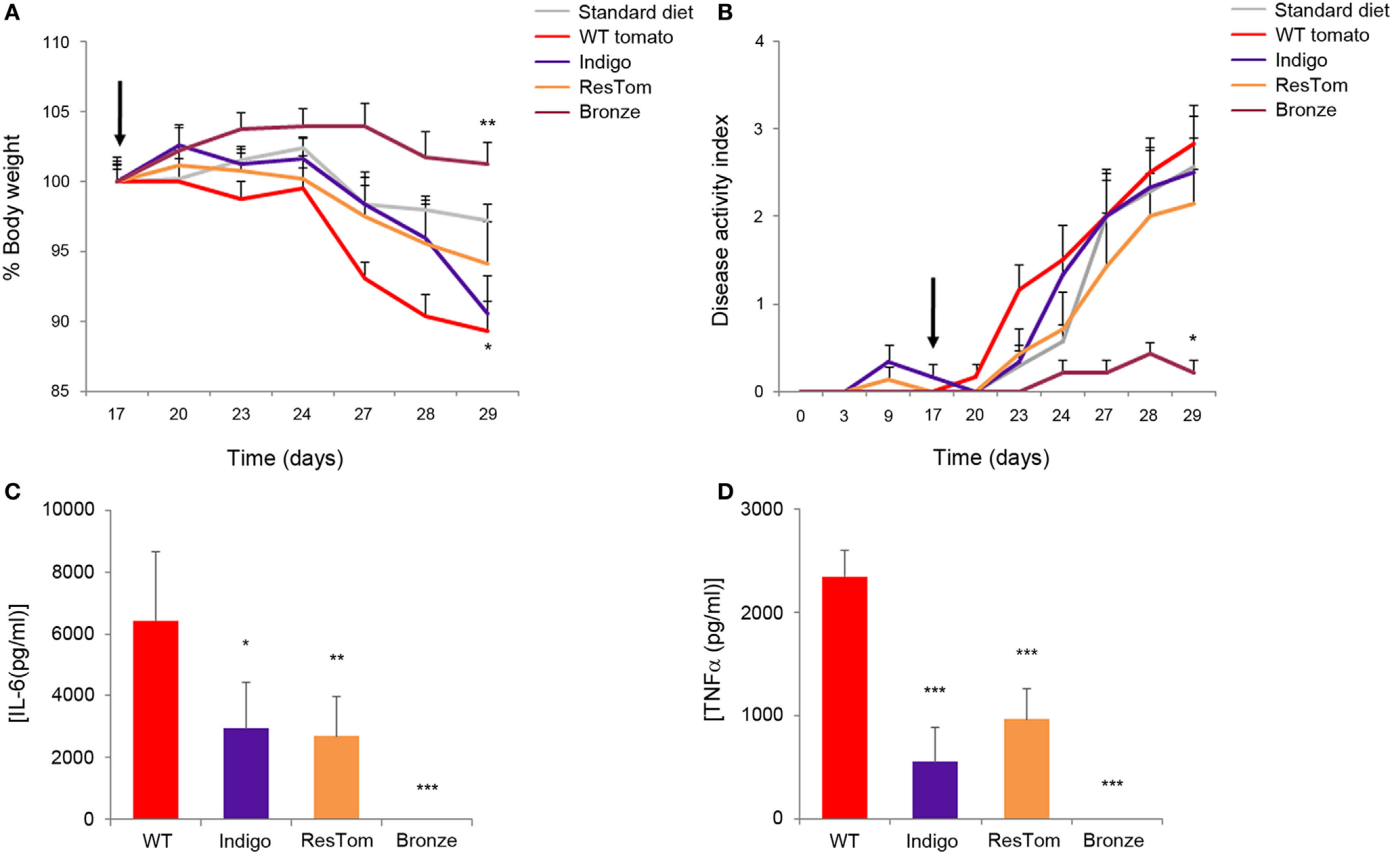
*In vivo* and *in vitro* effects of tomato fruit enriched in polyphenols. Average body weight **(A)** and disease activity index **(B)** of mice fed standard diet or diets enriched with 1% lyophilized tomatoes. Inflammation was induced by administration of 1% DSS to drinking water at day 17 (black arrow). Data are shown as means ± SEM (*n* = 5); * *P* < 0.05 (Student’s *t*-test). Production of pro-inflammatory cytokines IL-6 **(C)** and TNFα **(D)** in bone-marrow dendritic cells incubated with methanol extracts of different tomato fruit. Supernatants were collected 48 h after LPS stimulation and concentrations of cytokines were assessed by ELISA:* *P* < 0.05, ** *P* < 0.01, and *** *P* < 0.001 (Student’s *t*-test).

The effects of different diets enriched in polyphenols were assessed using a model of low, but prolonged, intestinal inflammation consisting of 1% DSS in drinking water administered to C57Bl/6 mice for 2 weeks (27). We used this DSS concentration to obtain a moderate chronic inflammation that could better mimic ulcerative colitis in a non-acute phase of the disease. Body weight, rectal bleeding, and general health conditions were monitored daily for each mouse (*n* = 5/group/three independent experiments) fed one of the five test diets. Administration of 1% DSS was sufficient to induce changes in the DAI (defined as the presence of blood in the stools, changes in stool consistency, and weight loss) and these changes varied significantly in response to different diets (Figures 3A,B). After 2 weeks of DSS treatment, all the mice were sacrificed and colons were measured since colon shortening is commonly used as a sign of inflammation (Figure S9 in Supplementary Material). Most strikingly, among DSS-treated mice, the colon length in the group receiving the Bronze tomato-supplemented diet was longer than in animals of all the other experimental groups and comparable to the length measured in control animals which did not receive DSS. Furthermore, in this group, stool consistency increased and intraluminal blood was reduced (Figure 3B). Histological examination revealed that the tomato-enriched diets were not able to protect from DSS-induced injuries, even though the epithelial erosion and intestinal crypt loss were less evident in tissue samples from mice fed with the Bronze-enriched diet compared with the other diets (Figure S9 in Supplementary Material).

### Production of Pro-Inflammatory Interleukins in DCs is Suppressed after Administration of Bronze Tomato Extract

To explore the immune-modulating properties of plant-derived polyphenols, we incubated murine BMDCs with methanol extracts of different tomatoes for 2 days prior to inflammatory stimulation with LPS. Results shown in Figures 3C,D and Figure S10 in Supplementary Material indicated that the extract of the Bronze tomato was able to suppress LPS-mediated production of inflammatory interleukins and TNF. None of the tomato extracts affected the phenotype or percentage of CD11c, MHCII^+^ DCs (Figure S10B in Supplementary Material). Extracts enriched in anthocyanins or stilbenoids were able to reduce the secretion of inflammatory cytokines to a lesser extent, suggesting that it was the combination of the specific polyphenols in Bronze tomatoes, rather than high levels of a single class of compounds, that was the predominant anti-inflammatory driving force in this cellular model.

Taken together, the gene expression profiles, the morphological analysis, and *in vitro* data on BMDC indicated that the diet supplemented with Bronze tomatoes reinforced the mucosal tolerance against undesired inflammation that leads to chronic inflammation and ulcerative colitis.

### Red Grape Skin-Enriched Diet Showed Similar Effects to the Bronze-Supplemented Diet on Chronic Inflammation in Mice

Among the different diets tested, which include different polyphenols, only the diet containing 1% Bronze tomatoes (approximatively 23 µg of anthocyanins, 150 µg of flavonols and 55 µg of stilbenoids per gram of complete diet, Table S7 in Supplementary Material) reduced the pathological symptoms of IBD in the 1% DSS, chronic inflammation, mouse model. Since Bronze tomato fruit contain a unique combination of these compounds, it is likely that functional interactions between these classes of polyphenols were responsible for the observed protection against the disease. To test this hypothesis, we searched among existing food sources for fruit or vegetables with a polyphenol profile similar to the one we had engineered in Bronze tomatoes.

**Figure 4:**
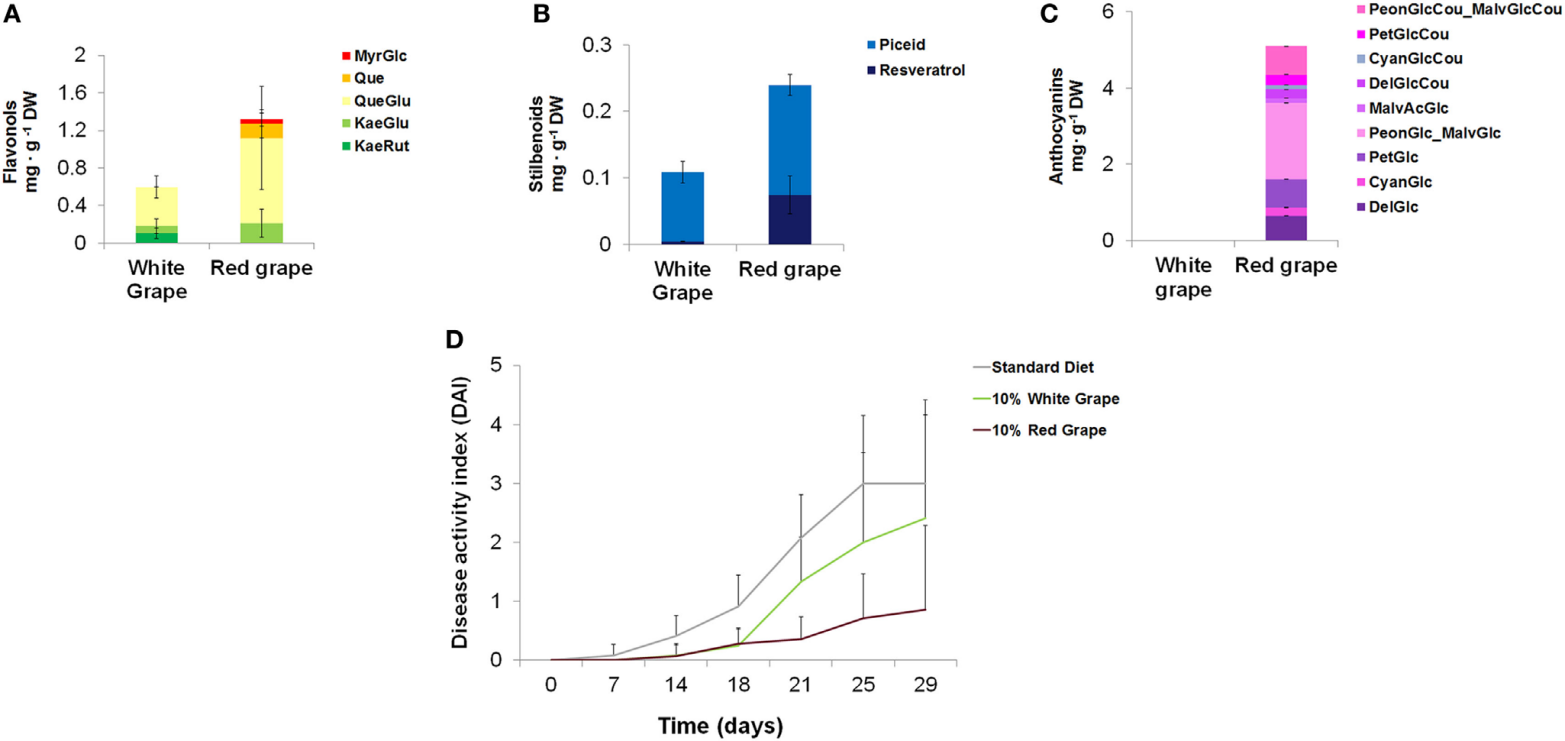
Quantification of different classes of polyphenols in white and red grape skin and effects on the intestinal inflammation in mice. Quantification by LC/MS analysis of flavonols **(A)**, stilbenoids **(B)**, and anthocyanins **(C)**, in white and black grape skin. Error bars show SEM (*n* = 3). MyrGlc, myricetin-glucoside; Que, quercetin; QueGlu, quercetin-glucoside; KaeGlu, kaempferol-glucoside; KaeRut, kaempferol-rutinoside; PeonGlcCou_MalvGlcCou, Peonidin-coumaroyl-glucoside_Malvidin-coumaroyl-glucoside; PetGlcCou, petunidin-coumaroyl-glucoside; CyanGlcCou, cyanidin-coumaroyl-glucoside; DelGlcCou, delphinidin-coumaroyl-glucoside; MalvAcGlc, malvidin-acyl-glucoside; PeonGlc-MalvGlc, peonidin-glucoside_malvidin-glucoside; PetGlc, petunidin-glucoside; CyanGlc, cyanidin-glucoside; DelGlc, delphinidin-glucoside. **(D)** Mice (*n* = 5) were divided into three groups of diet (standard diet and diets supplemented with 10% of white or black lyophilized grape skin). Diets started from day 0 and the treatment with 1% DSS in drinking water from day 14. Disease activity index was calculated for each group of diet.

Among fruits and vegetables commonly consumed in western countries, red grape accumulates stilbenoids, flavonols, and anthocyanins in the skin. White varieties of grape are unable to produce anthocyanins and, in general, contain lower levels of other polyphenols (Figure 4).

We formulated new diets enriched with either red or white grape skin and tested them on the same mouse model of IBD used for tomato-enriched diets. Since, after removal of fruit pulp and seeds, the concentration of flavonols and stilbenoids were lower (approximately 10-fold) than in whole Bronze tomatoes, we incorporated either lyophilized red or white grape skin at 10% w/w concentrations in the diets. Although the level of anthocyanins in the 10% red grape-enriched diet was 20-fold higher than in the diet supplemented with 1% Bronze tomato fruit, the contents of stilbenoids and flavonols were almost identical between the two supplements (Table S7 in Supplementary Material).

When tested on mice (*n* = 5) treated with 1% DSS to induce intestinal inflammation, the diet supplemented with red grape skin resulted in a substantial reduction of the DAI compared with the standard diet or to the diet supplemented with 10% white grape skin (Figure 4). Overall, colon length, reduction of BW loss, stool consistency, and rectal bleeding were indicative of reduced inflammation, as also observed for the diet supplemented with Bronze tomatoes. These results supported *in vitro* data on the anti-inflammatory properties of red grape skin on monocytic cells (28).

## Discussion

The near-isogenic tomato lines enriched in different polyphenols offer a powerful and innovative tool to compare the efficacy of plant bioactive compounds. Several preclinical and clinical studies have indicated that supplements and purified phytochemicals do not provide the same biological activity as whole foods rich in the same compounds (29). Isogenic plant-based foods with high levels of different bioactive compounds offer the opportunity to study these compounds and their potential additive and synergistic combinations in a whole food context against a wide range of chronic diseases (30). Here, we have shown the relevance of isogenic plant materials in assessing protective effects of dietary polyphenols on gut inflammatory responses. Bronze-enriched diets were able to reduce/delay the appearance of intestinal damage induced by DSS. Of note, there was a significant improvement in stool consistency, fecal blood content, and weight loss, even though Bronze-enriched diets could not provide full protection from DSS-mediated damage of intestinal epithelium, compared with standard or other tomato-enriched diets. In accordance with recent literature, a polyphenol-enriched diet might be involved in reduction of the release of inflammatory mediators and/or the promotion of production of proteins involved in tissue repair or those interfering with colonization by pathogens (31–34).

Metabolic engineering could be used to create a library of different functional foods fortified in specific sets of phytochemicals suitable for addressing a range of health needs. Such resources could be used to identify combinations of phytochemicals that can reduce or ameliorate inflammatory conditions through dietary intervention. Currently, the tomato lines are research tools only, but their development as functional foods remains an option for the future. Alternatively, a strategy based on analysis of data from the tomato-enriched diets, which allowed us to identify an existing food, red grape skin, as containing relatively high levels of polyphenols (~5-mg/g anthocyanins, ~1.2-mg/g flavonols, and ~0.3-mg/g stilbenoids) showed very similar protective effects to those of the diet containing 1% Bronze tomatoes. A similar functional interaction between different classes of polyphenols might explain some of the well-documented properties of red wine (35, 36). However, our results using red grape skin, if translated for application to humans (37), would involve consumption of more than 5 kg of fresh whole grape berries per day for a consumer aiming at protection against IBD. Alternatively, a single Bronze tomato fruit (approximately 60 g) would have a similar effect.

## Ethics Statement

This study was carried out in accordance with Directive 86/609 EEC enforced by Italian D.L. n. 116 1992. The protocol was approved by the Committee on the Ethics of Animal Experiments of Ministero della Salute––Direzione Generale Sanità Animale (Protocol no. 2012/00000923 A00: Eo_GINRC).

## Author Contributions

AS, EB, SS, EC, LH, MA, and MC performed the experiments. EB, MC, AS, and CM designed the experiments. AS, EB, SS, MA, GG, MC, AS, and CM analyzed and interpreted the data. AS, EB, MC, AS, and CM wrote the manuscript with the input from all the authors.

## Conflict of Interest Statement

The authors declare that the research was conducted in the absence of any commercial or financial relationships that could be construed as a potential conflict of interest.
