# Supplementary material for: Combined Dietary Anthocyanins, Flavonols, and Stilbenoids Alleviate Inflammatory Bowel Disease Symptoms in Mice
Source: Front Nutr. 2018 Jan 24;4:75. doi: 10.3389/fnut.2017.00075 (PMC5810255; doi:10.3389/fnut.2017.00075)
Supplement: Supplementary file 1 [file data_sheet_1.PDF]

SUPPLEMENTARY MATERIAL

Supplementary Methods

Grape polyphenols identification and characterization. Whole ripe grape fruits (cv. Negramaro black grape and cv. Verdeca white grape) were frozen in liquid nitrogen, freeze-dried and finely ground. Approximately 200 mg of powder were extracted twice with 5 ml 80% methanol at 4°C, overnight. Extracts were centrifuged at 4000 rpm for 20 min at 4°C and supernatants were collected. Supernatants were filtered through a 0.22 μ filter and then stored at -20°C until use. Extracts were run on a Shimadzu Nexera LC system attached to an IT ToF mass spectrometer using the same methods described for tomato polyphenols.

ORAC antioxidant capacity determination. Freeze-dried fruit powder (1g) was extracted twice in 80% methanol and the extracts used for antioxidant capacity assays. The Oxygen Radical Absorbance Capacity (ORAC) assay was carried out in 96 multi-well plates, in a final reaction mixture of 200 µl with 75mM phosphate buffer (pH 7.4). Antioxidant and fluorescein (final concentration 70 nM) solutions were introduced in each well and pre-incubated at 37 °C for 15 min. 2,2'-azobis-(2-methylpropionamidine) dihydrochloride (AAPH) solution (12mM) was rapidly added and fluorescence recorded every minute for 60 min, at excitation and emission wavelengths of 485 and 527 nm, respectively. The degree of antioxidant-mediated protection of samples was quantified using antioxidant Trolox as standard. Resulting ORAC values were expressed as µmol Trolox Equivalents (TE)/g DW.

TEAC antioxidant capacity determination. The Trolox Equivalent Antioxidant Capacity (TEAC) assays based on the scavenging ability of antioxidants to quench the radical cationic activity of 2,2'-azinobis (3-ethylbenzoithiazolone 6-sulphonate) ($ABTS^{\bullet+}$). The assay was performed as previously described (1) with some modifications. To generate the $ABTS^{\bullet+}$ radical cation, ABTS was dissolved in water (7 mM) and incubated with 2.45 mM potassium persulfate (final concentration) in the dark at room temperature for 12–16 h before use. For the calibration curve, the $ABTS^{\bullet+}$solution was diluted with water to an absorbance value of 0.70 (±0.02) at 734 nm and mixed with 20 µL of Trolox standard solutions (from 0 to 25 µM). The assay was performed with extracts from tomato fruits and absorbance was determined at 734 nm. Values were expressed as µmol Trolox Equivalents (TE)/g DW.

Determination of total polyphenols. The total polyphenol content in aqueous tomato extracts was determined according the Folin-Ciocalteau method, as previously described (2). All measurements were performed in triplicate and resulting values were expressed as mg gallic acid equivalent (GAE)/g of extract.

Carotenoid extraction and analysis. Carotenoids from ripe tomatoes were extracted and analyzed as previously described (3, 4).

Sugar extraction and analysis. Whole tomato fruits were frozen in liquid nitrogen, freeze-dried and finely ground. Approximately 200 mg of powder were extracted in 10 ml of distilled water and incubated, at 4°C, overnight. Extracts were centrifuged at 4000 rpm for 20 min, the supernatants collected, further centrifuged and filtered through a 0.22 μ filter. Extracts were diluted and analyzed with a Agilent 1100 Series HPLC system as previously described (5).

Microbial strains and culture conditions. The following probiotic strains *Lactobacillus rhamnosus* IMC501 and *Lactobacillus paracasei* IMC502 [SYNBIO100], *Lactobacillus rhamnosus* LRB, SP1, *Lactobacillus delbrueckii* SP5, *Lactobacillus plantarum* LPAL, *Lactobacillus casei* LC4P1, BGP3, *Lactobacillus acidophilus* LA3, *Bifidobacterium animalis* BLC1 were purchased by Sacco Srl.

Lactobacilli growth test. Methanol extracts from lyophilized tomato powder were filtered through 0.22 filter and added to MRS at a final concentration of 5 g/l. *Lactobacillus* cells used for the inoculum were previously cultured in MRS for 24 h at 37°C and centrifuged 9000 x g for 10 min. Cells were washed in sterile 20 mM of potassium phosphate buffer at pH 7.0. Each strain was inoculated at the final cell density of about 7 log CFU/mL in the media supplemented with tomato extracts. The incubation was carried out for 24 h at 37 °C and acidification of the media was determined by measurement of pH, compared to the control. Each trial was performed in triplicate.

DNA extraction from mice faecal samples and bacterial meta-genetic analysis. Three aliquots of faeces collected from each animal were pooled together for DNA extraction. After homogenization in RNA later, faecal samples were mixed 1:1 with distilled water in sterile plastic pestle. The homogenate was subjected to mechanical disruption in a FastPrep® instrument (BIO 101) and total DNA was extracted with a FastDNA® Pro Soil-Direct Kit (MP Biomedicals, CA., USA), according to the manufacturer's instructions. An aliquot of about 300 μl of each faecal sample was diluted in 1

ml PBS-EDTA (phosphate buffer 0.01 M, pH 7.2, 0.01 M EDTA). After centrifugation (14,000 × *g* at 4°C for 5 min), the pellet was washed two times to decrease the content of PCR inhibitors. The resulting pellet was resuspended in 300 µl of PBS-EDTA and used for DNA extraction with a FastPrep. DNA extracted from faecal samples was used as the template for the sequencing of the 16S rRNA gene by Illumina MiSeq diversity analyses, which was carried out at the Genomix4Life S.r.l. (Baronissi, Salerno, Italy).

Taxonomic Identification. The sequenced reads were processed through de-noising and chimera detection. De-noising was performed through the following steps: (i) merging together the forward and reverse reads using the PEAR Illumina paired-end read merger (6); (ii) reading the runs through an internally developed quality trimming algorithm that truncates reads having an average quality higher than 25; (iii) grouping reads by using the USEARCH (7) algorithm (prefix dereplication) into clusters (4% dissimilarity among sequences of the same cluster), so that each sequence of shorter length to the centroid sequence must show a 100% match to the centroid sequence for the length of the sequence; and (iv) operational taxonomic unit (OTU) selection by using the UPARSE OTU selection algorithm (8) to classify the large number of clusters into OTUs. Following de-noising, the selected OTUs were chimera checked using the UCHIME software executed in de novo mode (9). Each trimmed read was mapped to its corresponding non chimeric cluster using the USEARCH global alignment algorithm (7). Each sequence in a cluster was then aligned to the consensus sequence. Each sequence was corrected base by base in order to remove noise. Analysis of microbial diversity was finally performed by running the centroid sequence from each cluster against the USEARCH algorithm, using a database of high-quality sequences derived from the NCBI database. Lastly, the output was analyzed using an internally developed python program that assigns taxonomic information to each sequence.
The percentage of each bacterial OTU was analyzed individually for each sample, providing relative abundance information among the samples based on the relative numbers of reads within each (10).

Statistical analyses. Analysis of variance (ANOVA) was carried out on transformed data, followed by separation of means with Tukey's honestly significant difference (HSD) test, using the statistical software Statistica 7.0 for Windows (StatSoft, Vigonza, Italy). Weighted and unweighted UniFrac distance matrices and OTU tables were used to perform ADONIS and ANOSIM statistical tests through the compare_category.py script of QIIME to verify the microbial populations.

Host gene expression profiling. Total RNA was isolated from colon samples using TRIzol® (Thermo Fisher Scientific, MA, USA) according to manufacturer's instructions. Total RNA (500ng) was reverse transcribed with the High Capacity cDNA Reverse Transcription kit (Thermo Fisher Scientific, MA, USA) by using random primers for cDNA synthesis. A Predesigned 96-well panel of genes for use with SYBR® Greenwell "Colitis, Ulcerative Tier 1 M96" (BioRad) was used to obtain gene expression profiles. Real-time analysis was run on CFX96 System (Biorad Laboratories, CA, USA) and the expression of all target genes was calculated relative to GAPDH expression using the $\Delta\Delta Ct$ method.

Correlation analyses. The co-occurrence of genera across samples was based on Pearson's correlation analysis ($P<0.05$), clustering and visualization of the correlations were obtained with "corrplot" package in R, version 3.2.2. For the Spearman's ranked correlation analysis on the microbial genera and polyphenols in diets, significant correlations were considered with a false discovery rate (FDR)-adjusted P-value <0.05.

ELISA. Cell culture supernatants were analyzed for IL-1α, IL-1β and IL-12 release in triplicate, using ELISA kit (R&D Systems, Minneapolis, MN, USA) following manufacturer' instructions.

Cytofluorimetric analysis. BMDCs were stained with CD11c-PE and MHCII-APC (Miltenyi Biotec, Begisch Gladbach, Germany). Flow Cytometer acquisition was performed using NAVIOS (Beckman, CA, USA).

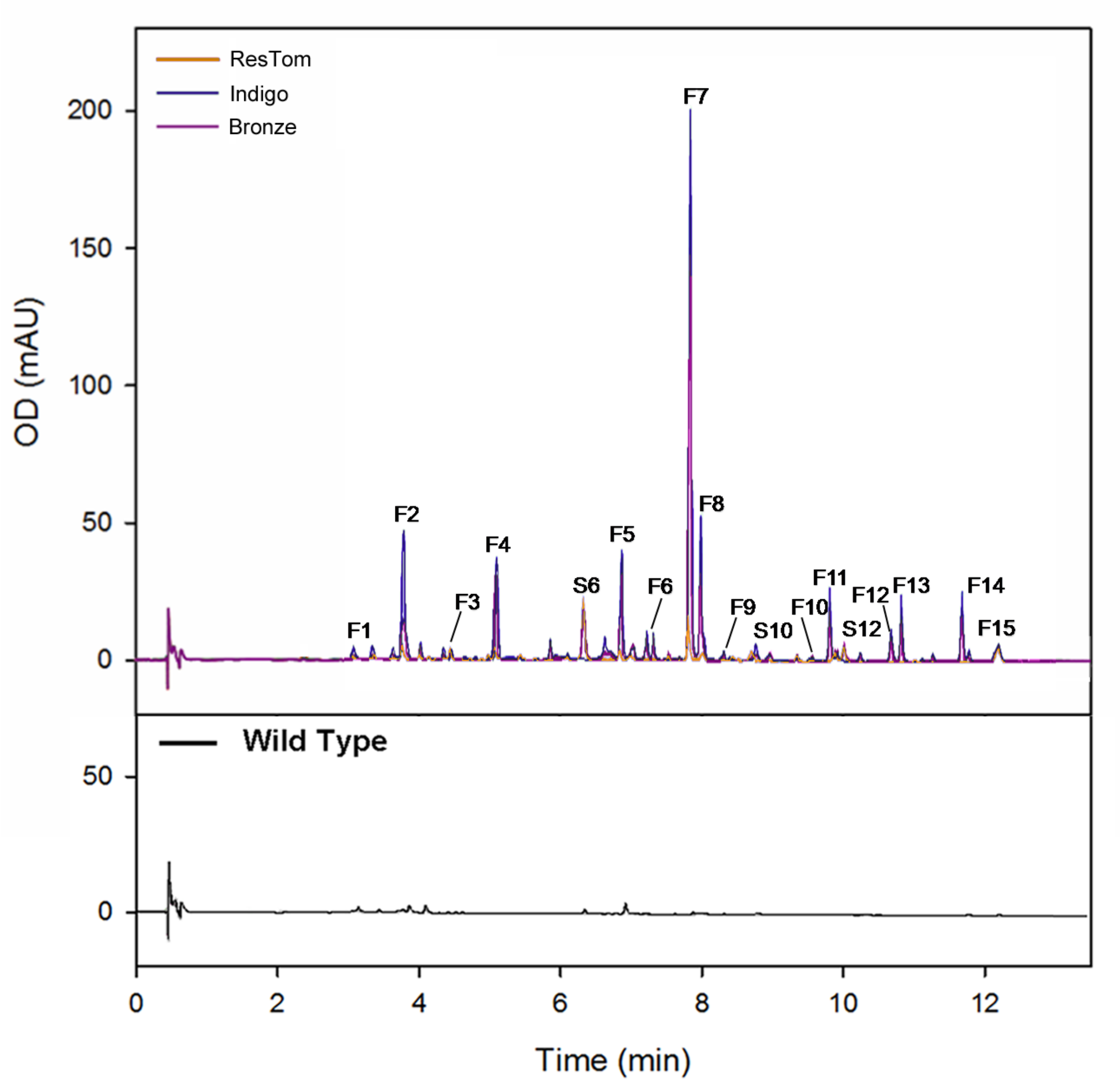

Supplementary Figure 1

Identification of flavonols and other polyphenols in different tomato fruit. LC/MS analysis of polyphenols detected at 350 nm wavelength in methanol extracts of ripe tomato fruit. Details of peaks wavelengths, retention times and fragmented ions specific for each compound are reported in Supplementary Table 1. The different tomato lines are indicated as follows: 'ResTom' (yellow line); 'Indigo' (blue line;); 'Bronze' (purple line).

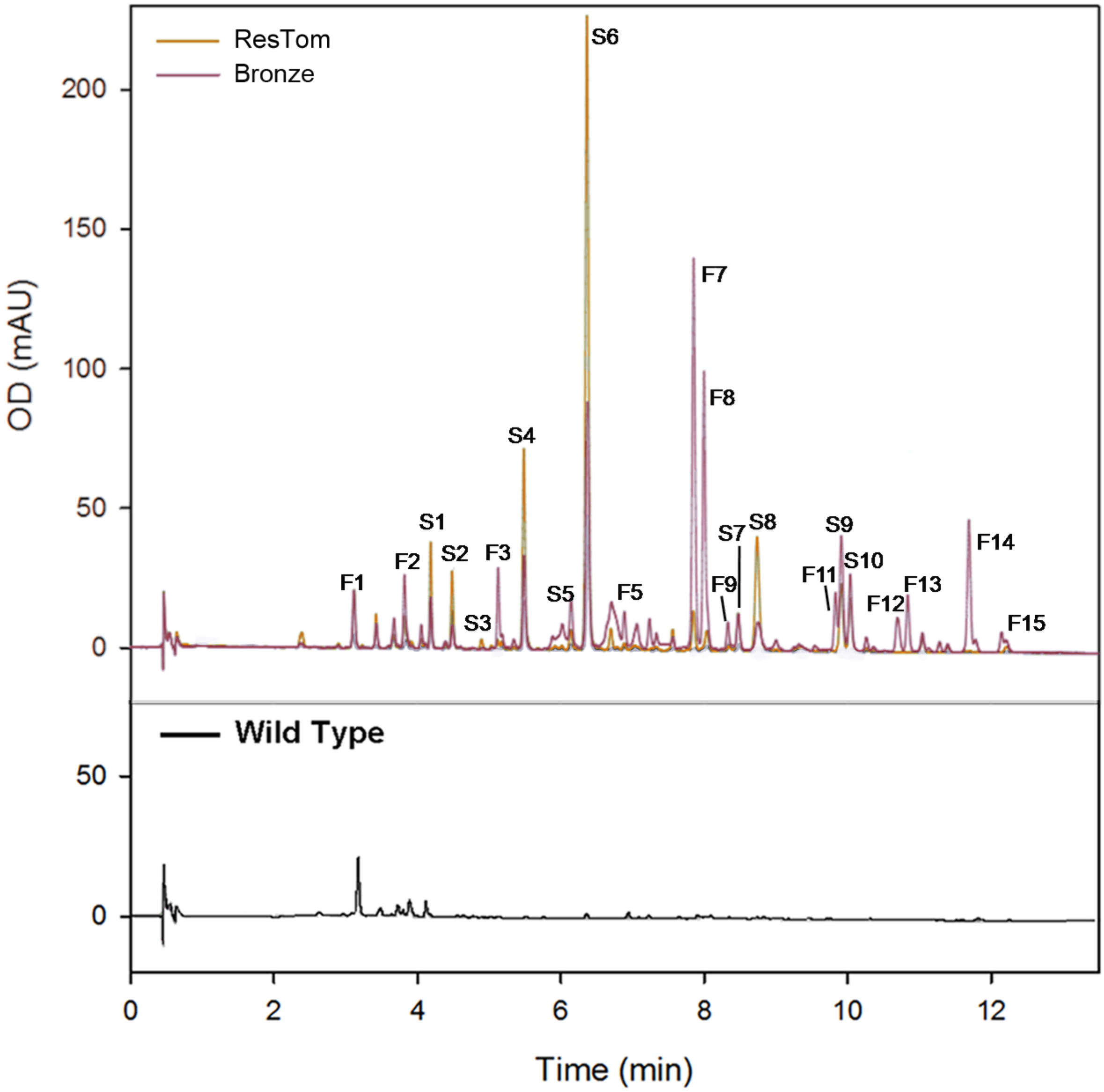

Supplementary Figure 2

Identification of stilbenes in different tomato fruits. LC/MS analysis of different stilbenes detected at 306 nm wavelength in methanol extracts of ripe fruits of different tomato lines. Details of peaks wavelengths, retention times and fragmented ions specific for each compound are reported in Supplementary Table 2. The different tomato lines are indicated as Supplementary Figure 1.

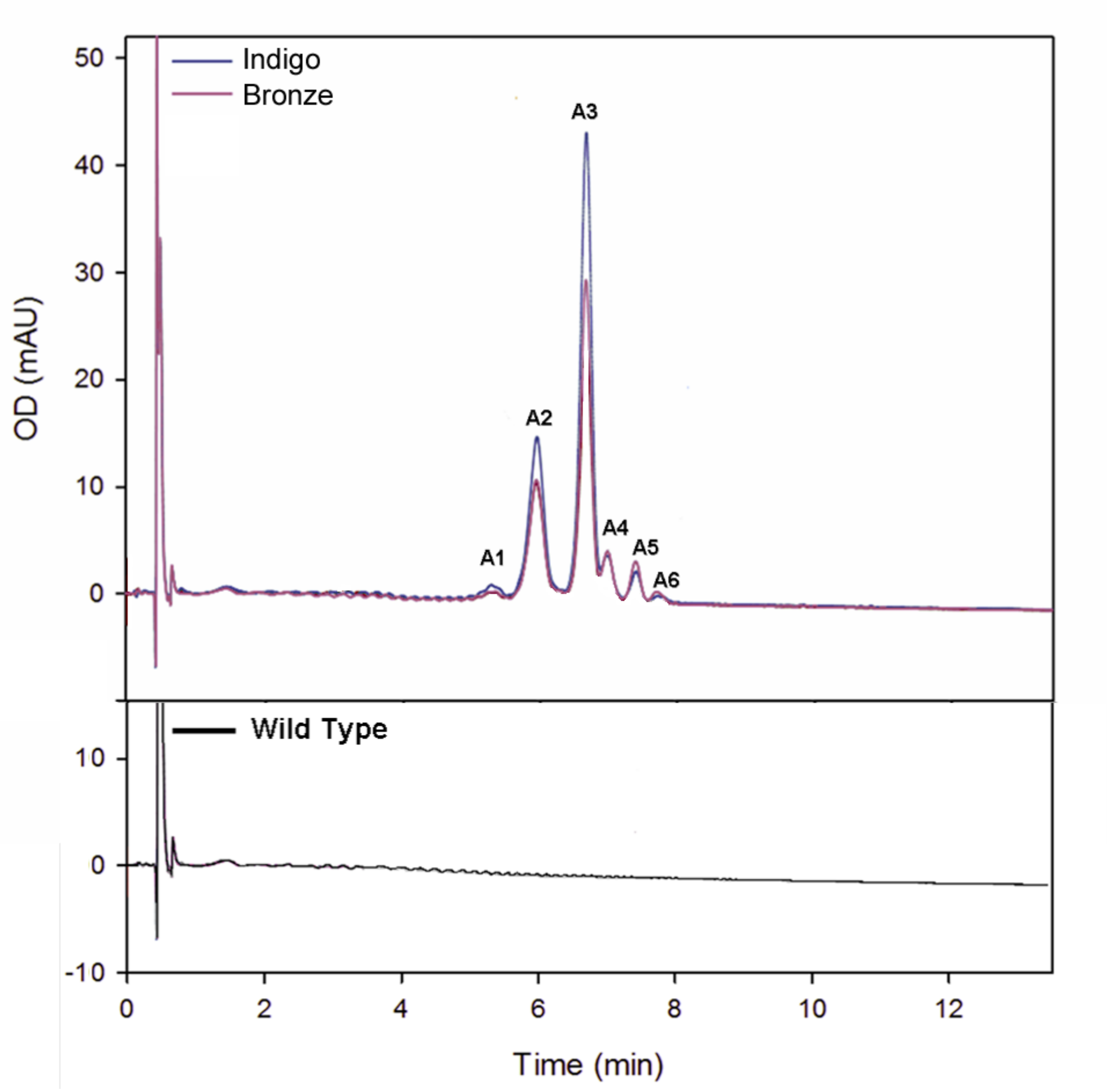

Supplementary Figure 3

Identification of anthocyanins in different tomato fruits. LC/MS analysis of anthocyanins detected at 525 nm wavelength in methanol extracts of ripe fruits of different tomato lines. Details of peaks wavelengths, retention times and fragmented ions specific for each compound are reported in Supplementary Table 3. The different tomato lines are indicated as in Supplementary Figure 1.

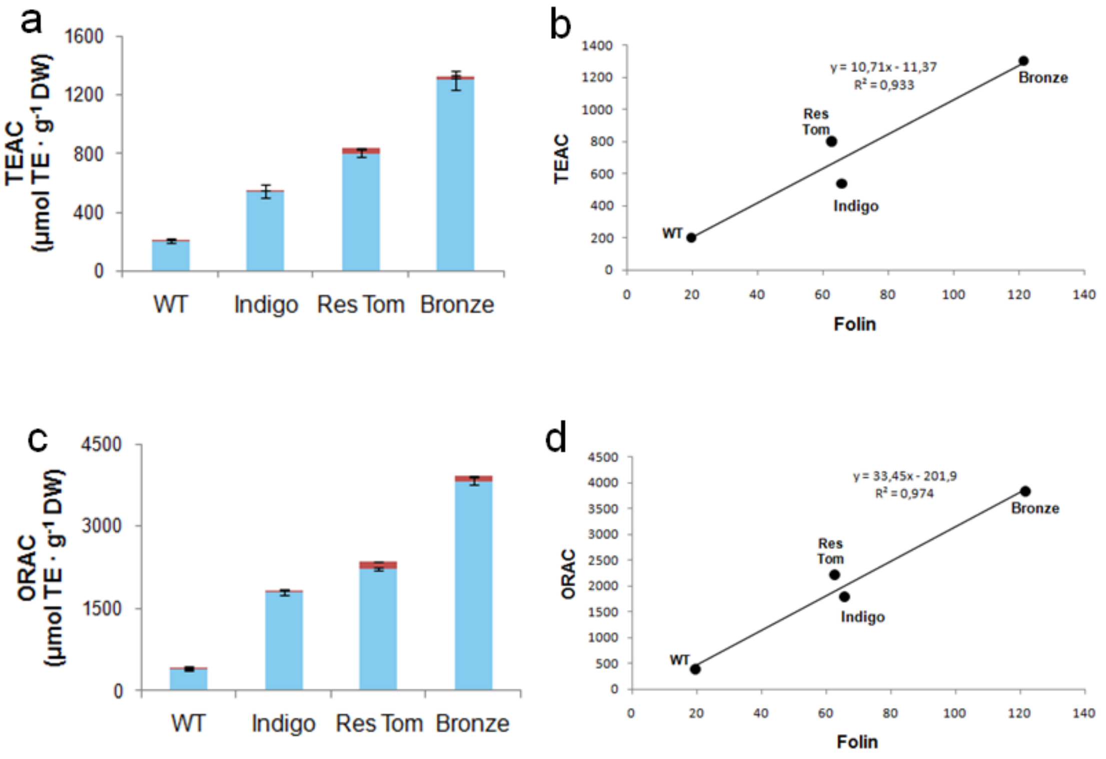

Supplementary Figure 4

Antioxidant capacity and correlation with total content of polyphenols. Antioxidant capacity of ripe fruit was measured in both hydrophilic (blue histograms) and lipophilic (red histograms) fractions by trolox equivalent antioxidant capacity (TEAC) assay (a) and oxygen radical capability (ORAC) assay (c). Hydrophilic antioxidant capacity was correlated with total polyphenol content measured by Folin-Ciocalteau assay (b, d). Values are shown as mean ± s.e.m. (n=3).

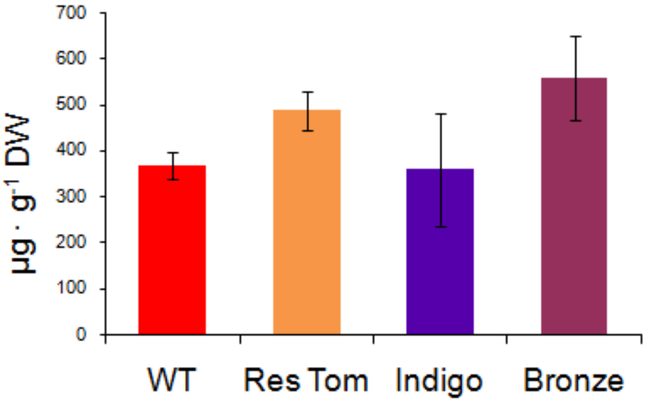

Supplementary Figure 5

Total content of carotenoids in different tomato lines. The content of main carotenoids classes detected in ripe fruits of different tomato lines was determined by HPLC analysis. Values are shown as mean ± s.e.m (n=3). No significant differences were found among different tomato lines.

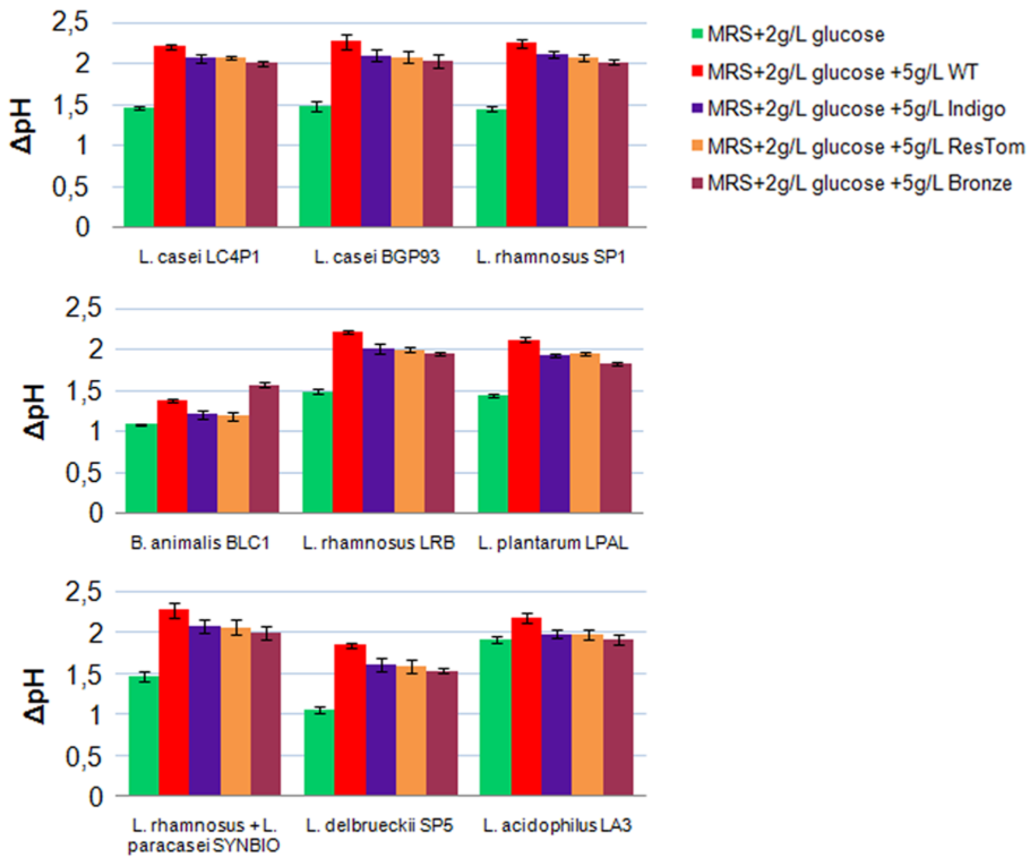

Supplementary Figure 6

Bacterial cell growth measured by medium acidification (ΔpH) of probiotic strains belonging to the genera *Lactobacillus* (L.) and *Bifidobacterium* (B.). Bacteria were cultivated in MRS medium containing 2 g/l of glucose and supplemented with extracts of different tomato lines.

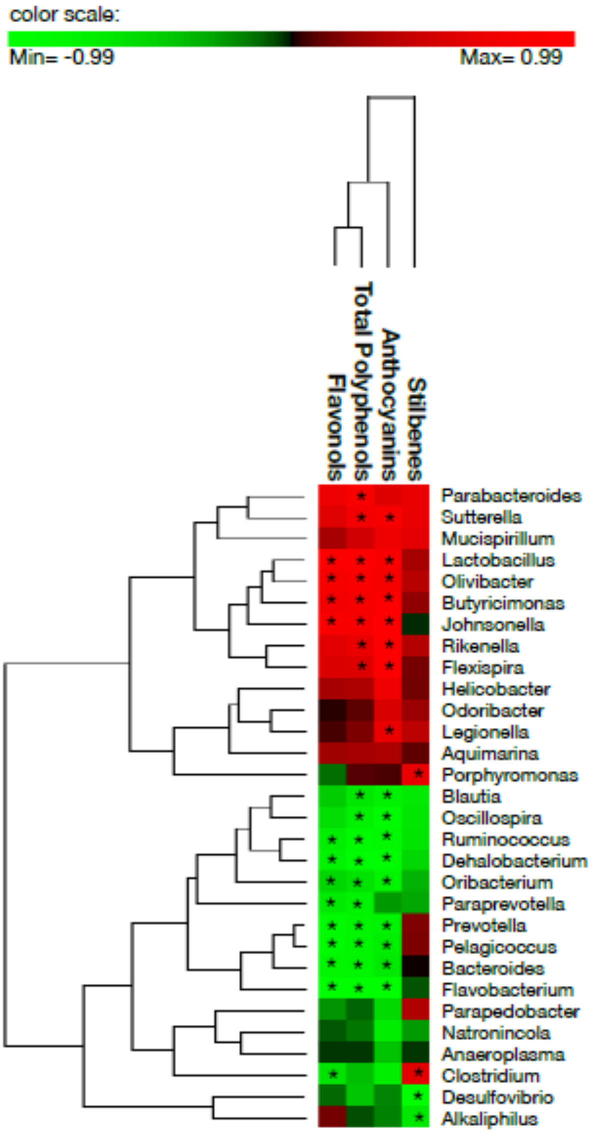

Supplementary Figure 7

Correlation analysis between dietary polyphenols and faecal microbial genera. Heatmap comparing the degree of association between the 30 most abundant microbial genera of the mouse microbiome and dietary polyphenols (flavonols, anthocyanins, stilbenes and total polyphenols), carried out by Spearman's correlations. The correlation values range from -0.99 (negative correlations, indicated in green) to 0.99 (positive correlations, in red). Hierarchical clustering is based on Euclidean distance and ward linkage. Asterisks indicate significant correlations after p-value correction (FDR < 0.05).

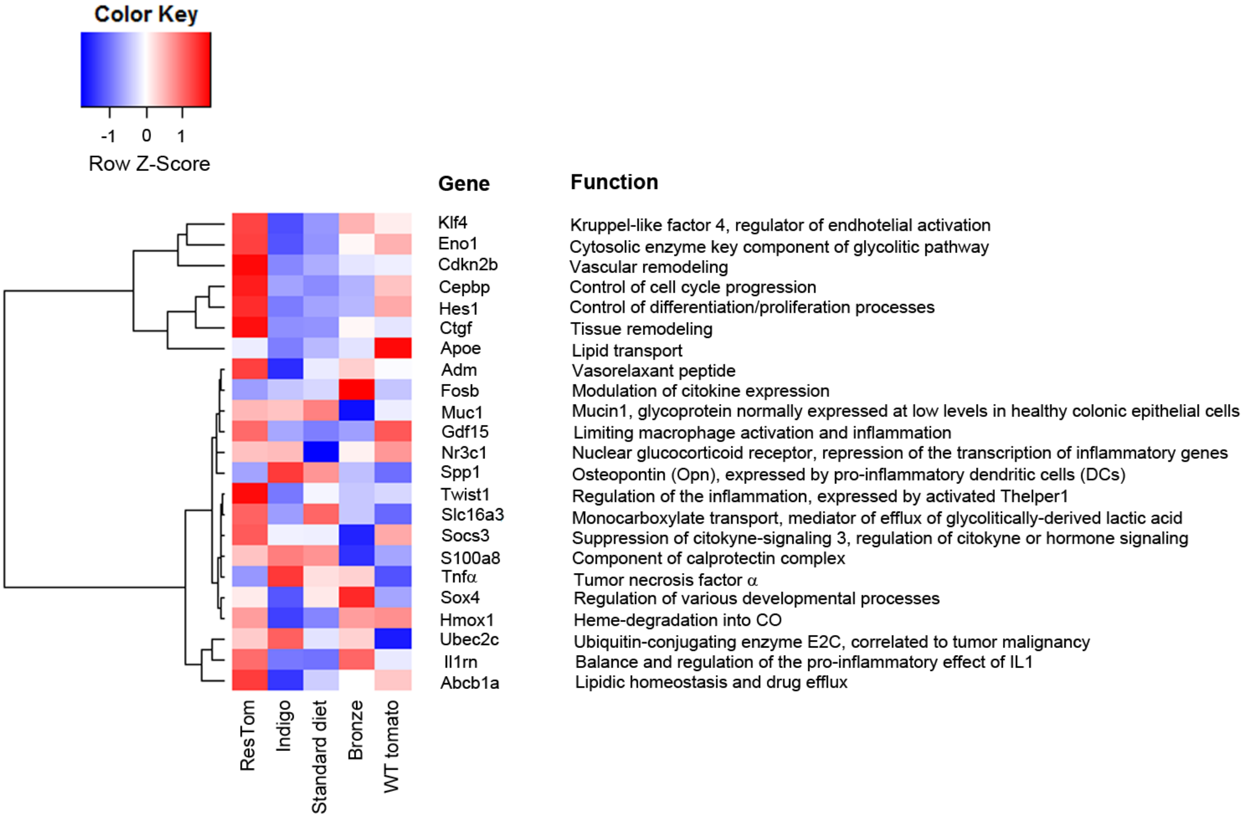

Supplementary Figure 8

Effects of polyphenol-enriched tomato diets on the host's colon gene expression. Heatmap comparing the gene expression profile induced in the host colon by different tomato-based diets supplemented for 2 weeks. Hierarchical clustering is based on Euclidean distance and ward linkage. Expression values, recorded as fold change, are represented in scaled expression, denoted as Row Z-Score and plotted in blue-red scale, with red indicating high expression and blue indicating low expression.

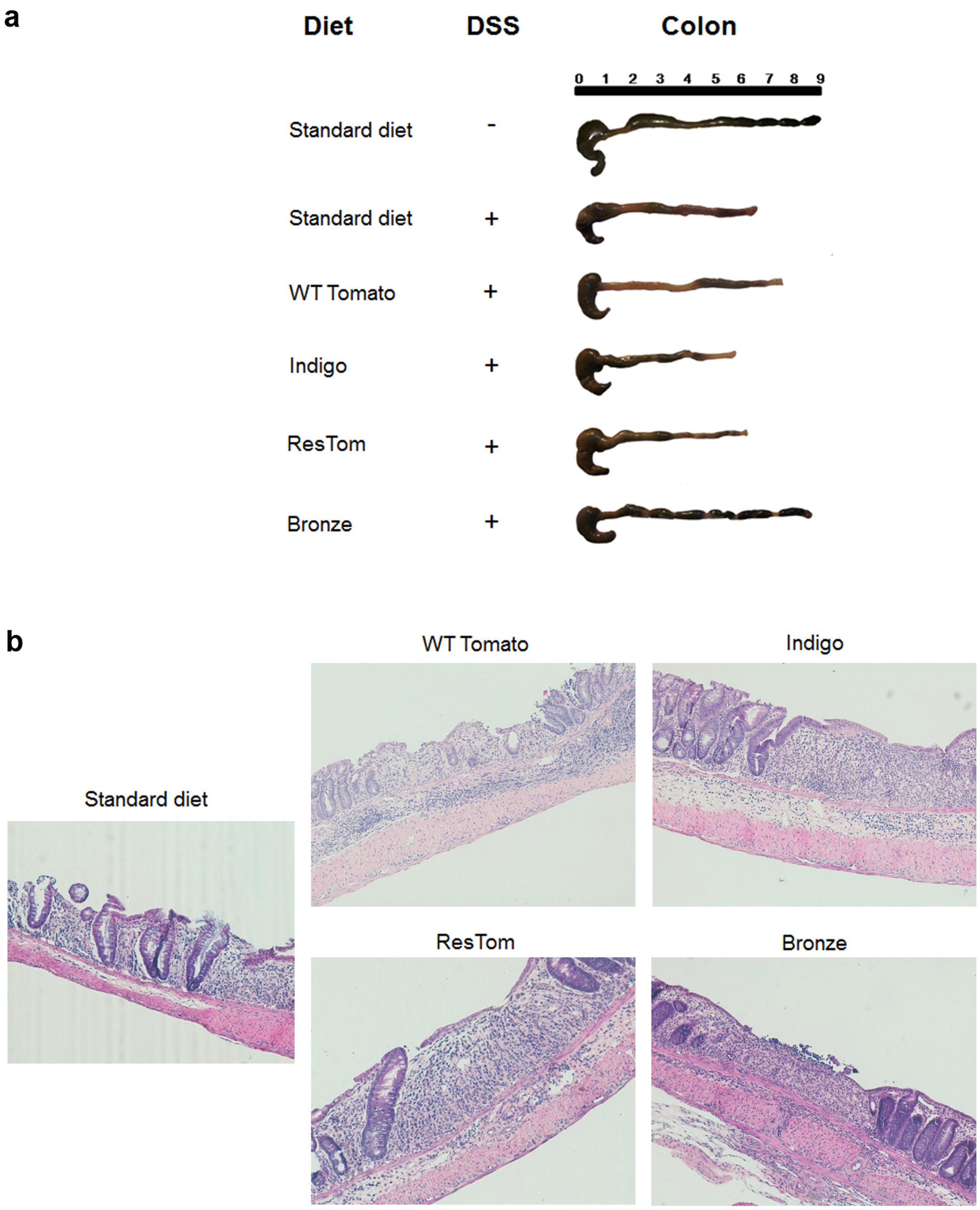

Supplementary Figure 9

Effect of polyphenol-enriched tomato diets on the colon morphology in the IBD mouse model. a) Morphological changes in the colons of mice fed different tomato-based diets and treated with 1% DSS. Representative organs for each group showing differences in colon length, stool consistency and intraluminal blood. b) Histological sections of distal colon from each group of diet after 1% DSS treatment.

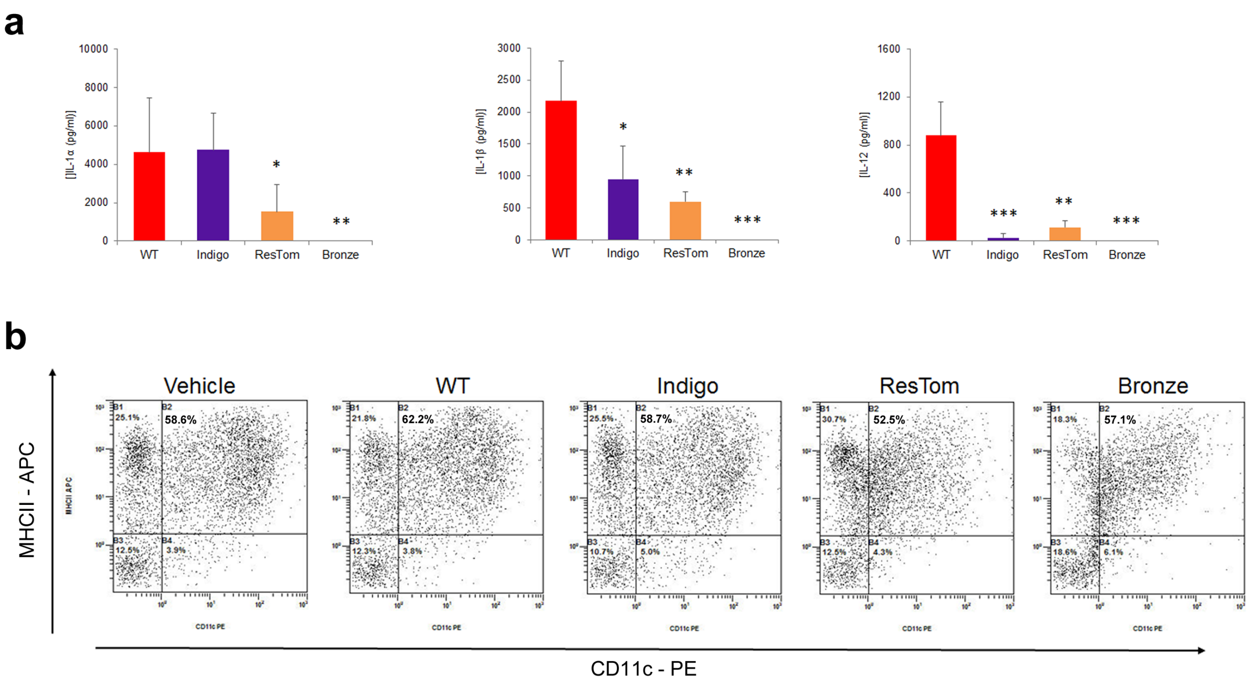

Supplementary Figure 10

Tomato polyphenols administration reduces LPS-mediated interleukins production in BMDCs.

a) Cell supernatants were collected 48h after LPS administration and used to assess IL-1α, IL-1β and IL-12 by ELISA. **b**) Surface expression of BMDC CD11c and MHCII treated with tomato extracts.

Supplementary Table 1. Peak identification of flavonols and other polyphenols from LC/MS data.

Peak	Abbreviation	Compound	λmax (nm)	Retention time (min)	[M+H]+/[M+Na]+ (m/z)	Detected fragments
F1-F2	CouGlc	Coumaroyl glucoside	350	3,1; 3,93	349,09	349,09
F2	CGA	Chlorogenic acid	325	3,82	355,10	355,1
F3	RutGlc	Rutin glucoside	350	4,50	773,1	773,1 (611,1; 303,05)
F4	KaeGlcGlc	Kaempferol diglucoside	350	5,04	611,16	611,16 (449,11; 287,05)
F4	KaeRutGlc	Kaempferol-rutinoside-glucoside	350	5,13	757,22	757,22 (449,11; 287,05)
F5	Rut	Rutin	350	6,98	611,16	611,16 (465,10; 303,05)
F6	MeMyrGlc	Methyl-myricetin-glucoside	350	10,73	809,19	809,19 (495,11; 333,06)
F7	KaeRut	Kaempferol rutinoside	350	7,87	595,17	595,17 (287)
F8-F12	KaeGlc	Kaempferol glucoside	350	8,06; 10,74	449,11	449,11 (287,05)
F9	IsorhamRut	Isorhamnetin-rutinoside	350	8,21	625,18	625,18 (479,12; 317,06)
F10	NarGlc	Naringenin glucoside	368	9,39	457,11	457,11 (273,07)
F11	MyrCouRut	Myricetin-coumaroyl-rutinoside	350	10,00	795,17	795,17 (481,09; 319,04)
F13	QueCouRut	Quercetin-coumaroyl-rutinoside	350	10,91	779,18	779,18 (465,10; 303,05)
F14	KaeCouRut	Kaempferol-coumaroyl-rutinoside	350	11,79	763,1	763 (449,1; 287,05)
F15	NarCh	Naringenin chalcone	365	12,22	273,08	273,08

Supplementary Table 2. Peak identification of stilbenes from LC/MS data.

Peak	Abbreviation	Compound	λmax (nm)	Retention time (min)	[M+H]+/[M+Na]+ (m/z)	Detected fragments
S1	ResGlcGlc1	Resveratrol-glucoside isoform 1	306	4,19	575,17	575,17 (413,12; 229,08)
S2	ResGlcGlc2	Resveratrol-glucoside Isoform 2	306	4,49	575,17	575,17 (413,12)
S3	ResGlcGlc3	Resveratrol-glucoside Isoform 3	306	4,90	575,17	575,17 (413,12)
S4	ResGlcGlc4	Resveratrol-glucoside Isoform 4	306	5,49	437	437
S5	ResGlcGlc5	Resveratrol-glucoside Isoform 5	306	6,14	575,17	575,17 (413,12)
S6	Piceid	Piceid	306	6,38	391,14	391,14 (229,08)
S7	MeResGlcGlc	Methyl-resveratrol-diglucoside	306	8,48	589,19	589,19 (405,15; 243,09)
S8	Res	Resveratrol	306	8,75	229,08	229,08
S9	MeResGlc	Methyl-resveratrol-glucoside	306	9,92	427,14	427,14 (243,09)
S10	ResGlcCaf	Resveratrol-glucoside-caffeate	306	10,04	553,17	553,17 (391; 228,25)

Supplementary Table 3. Peak identification of anthocyanins from LC/MS data.

Peak	Abbreviation	Compound	λmax (nm)	Retention time (min)	[M+H]+/[M+Na]+ (m/z)	Detected fragments
A1	DelGlcCafRut	Delphinidin-glucoside-caffeoyl-rutinoside	525	5,3	935,25	935,24 (787,23; 469,13; 303,05)
A2	DelGlcCouRut	Delphinidin-glucoside-coumaroyl-rutinoside	525	6	919,25	919,25 (757,19; 465,10; 303,05)
A3	PetGlcCafRut	Petunidin-glucoside-caffeoyl-rutinoside	525	6	949,26	949,26 (787,21; 479,12; 317,06)
A3	PetGlcCouRut	Petunidin-glucoside-coumaroyl-rutinoside	525	6,72	933,27	933,26 (771,21; 479,12; 317,06)
A4	PetGlcFerRut	Petunidin-glucoside-feruloyl-rutinoside	525	7	963,27	963,27 (801,22; 479,12; 317,06)
A5	MalGlcCouRut	Malvidin-glucoside-coumaroyl-rutinoside	525	7,4	947,28	947,28 (785,23; 493,13; 331,08)
A6	MalGlcFerRut	Malvidin-glucoside-feruloyl-rutinoside	525	7,45	977,29	977,29 (815,24; 493,13; 331,08)

Supplementary Table 4. Reads number, number of identified species, Shannon diversity index and Chao 1 mean of the microbiota of mice fed different diets (n=5; A: standard diet; B: wild type tomato; C: Indigo; D: ResTom; E: Bronze.

Sample	Number Reads PF	Number of species identified	Shannon Species diversity	Chao 1 Mean	Chao 1 95% CI Lower Bound	Chao 1 95% CI Upper Bound	Chao 1 SD (analytical)
A1	482169	483	2.285	579,89	527,95	664,64	34,04
A2	503604	449	2.047	704,59	646,55	796,39	37,43
A3	550214	467	2.225	788,76	722,51	892,09	42,41
A4	595934	488	2.261	856,58	784,38	967,65	45,89
A5	242512	316	2.129	904,94	830,2	1018,69	47,24
B1	275539	361	2.285	523,02	469,16	612,74	35,69
B2	265289	354	2.161	631,81	574,25	724,06	37,37
B3	277447	379	2.283	708,26	648,07	802,86	38,69
B4	447517	404	2.268	751,33	692,5	842,79	37,6
B5	422382	434	1.948	776,56	721,52	861,71	35,09
C1	272824	443	2.312	498,69	453,13	574,29	30,12
C2	270082	354	2.034	605,17	556,97	681,96	31,2
C3	260583	367	2.097	669,16	620,2	745,8	31,4
C4	357244	396	2.139	729,38	674,49	814,76	35,09
C5	193322	330	2.146	778,23	719,51	868,7	37,35
D1	306059	367	2.064	547,68	493,31	637,48	35,86
D2	380938	405	2.130	650,65	593,01	743,38	37,5
D3	413790	407	2.089	707,16	651,73	794,78	35,73
D4	407723	429	2.042	765,89	705,45	860,43	38,75
D5	394342	445	2.089	820,72	754,36	923,37	42,3
E1	296675	376	2.170	516,32	462,62	605,83	35,59
E2	375463	439	2.241	635,53	578,66	725,96	36,78
E3	319637	339	2.050	748,64	675,84	862,03	46,57
E4	315227	392	2.302	835,65	752,3	963,07	52,8
E5	359176	382	2.320	892,58	806,96	1021,06	53,72

Supplementary Table 5. Changes in the whole microbiome induced by different diets. (file excel)

Supplementary Table 6. Pearson's correlation analysis between genera of the microbiota of mice fed different diets. Significant positive and negative correlations are shown in red and blue, respectively.

	Standard diet			WT tomato Diet			ResTom Diet			Indigo Diet			Bronze Diet		
**Genus**	**Significant correlation**	**Pearson r correlation**	**P value**	**Significant correlation**	**Pearson r correlation**	**P value**	**Significant correlation**	**Pearson r correlation**	**P value**	**Significant correlation**	**Pearson r correlation**	**P value**	**Significant correlation**	**Pearson r correlation**	**P value**
**Parabacteroides**	Oscillospira	0.9077	0.0332	Olivibacter	0.8955	0.0399	Oribacterium	0.9132	0.0303	Aquimarina	0.9407	0.0172	Bacteroides	-0.9296	0.0222
	Butyricimonas	0.9583	0.0102	Porphyromonas	0.9595	0.0097									
	Sutterella	0.9043	0.035	Sutterella	0.9787	0.0037									
				Ruminococcus	-0.9797	0.0035									
				Clostridium	-0.9322	0.021									
**Blautia**	Odoribacter	0.9063	0.0339	Desulfovibrio	0.8854	0.0458	Parapedobacter	0.899	0.0379	Flavobacterium	-0.9207	0.0265	Odoribacter	0.9246	0.0245
				Ruminococcus	0.8919	0.042	Natronincola	0.9124	0.0307				Desulfovibrio	0.9049	0.0347
				Prevotella	-0.9294	0.0223	Rikenella	-0.8876	0.0445				Oscillospira	0.992	0.0009
				Paraprevotella	-0.8936	0.041							Ruminococcus	0.9817	0.003
													Legionella	0.8968	0.0392
													Dehalobacterium	0.9947	0.0005
													Sutterella	-0.947	0.0145
**Prevotella**	Olivibacter	-0.9012	0.0367	Blautia	-0.9294	0.0223	Porphyromonas	0.9962	0.0003	Bacteroides	0.8958	0.0397	Flavobacterium	0.9161	0.0288
				Desulfovibrio	-0.8917	0.0421	Paraprevotella	0.9515	0.0127	Odoribacter	0.9506	0.0131	Clostridium	-0.8898	0.0432
										Helicobacter	-0.989	0.0014			
										Mucispirillum	-0.9928	0.0007			
										Flexispira	-0.9191	0.0273			
**Helicobacter**	Mucispirillum	0.9828	0.0027	Mucispirillum	0.9463	0.0148	Lactobacillus	0.8936	0.041	Mucispirillum	0.9858	0.002	Flexispira	0.9961	0.0003
	Flexispira	0.9834	0.0026	Flexispira	0.9969	0.0002	Olivibacter	0.9502	0.0132	Flexispira	0.9402	0.0174	Legionella	-0.9241	0.0248
	Oribacterium	0.9145	0.0296	Legionella	0.9885	0.0015	Flexispira	0.9544	0.0116	Prevotella	-0.989	0.0014			
	Natronincola	0.967	0.0072	Aquimarina	0.944	0.0158	Bacteroides	-0.8868	0.0449	Odoribacter	-0.9046	0.0348			
	Oscillospira	-0.9503	0.0132	Bacteroides	-0.8923	0.0417				Paraprevotella	-0.8864	0.0452			
	Dehalobacteriun	-0.8897	0.0432												
**Lactobacillus**							Helicobater	0.8936	0.041	Porphyrimonas	-0.9561	0.011	Sutterella	0.9664	0.0073
							Flexispira	0.9185	0.0276				Ruminococcus	-0.902	0.0363
							Aquimarina	0.9095	0.0322				Pelagicoccus	-0.9254	0.0242
**Bacteroides**	Porphyromonas	0.9259	0.0239	Helicobacter	-0.8923	0.0417	Helicobacter	-0.8868	0.0449	Prevotella	0.8958	0.0397	Parabacteroides	-0.9296	0.0222
	Paraprevotella	0.9472	0.0144	Flexispira	-0.9104	0.0318	Ruminococcus	-0.9326	0.0208	Odoribacter	0.9472	0.0144			
	Pelagicoccus	-0.9844	0.0023	Clostridium	-0.8836	0.0469	Mucispirillum	-0.9872	0.0017	Mucispirillum	-0.9251	0.0243			
	Oribacterium	-0.8837	0.0468	Aquimarina	-0.9317	0.0212									
**Odoribacter**	Blautia	0.9063	0.0339	Johnsonella	0.9247	0.0245	Desulfovibrio	-0.89	0.0431	Prevotella	0.9506	0.0131	Blautia	0.9246	0.0245
	Mucispirillum	-0.9246	0.0246	Porphyromonas	-0.9658	0.0076	Natronincola	-0.8864	0.0452	Bacteroides	0.9472	0.0144	Desulfovibrio	0.8973	0.0389
	Flexispira	-0.8947	0.0404	Sutterella	-0.9066	0.0338				Mucispirillum	-0.9603	0.0094	Oscillospira	0.8982	0.0384
	Natronincola	-0.9371	0.0188							Helicobacter	-0.9046	0.0348	Ruminococcus	0.8885	0.0439
													Legionella	0.9457	0.0151
													Dehalobacterium	0.9079	0.0331
**Olivibacter**	Prevotella	-0.9012	0.0367	Parabacteroide	0.8955	0.0399	Helicobacter	0.9502	0.0132	Butyricimonas	0.9197	0.027			
				Sutterella	0.943	0.0162	Sutterella	0.8849	0.0461						
							Flexispira	0.8956	0.0398						
**Desulfovibrio**				Blautia	0.8854	0.0458	Odoribacter	-0.89	0.0431	Natronincola	0.8965	0.0393	Odoribacter	0.9049	0.0347
				Ruminococcus	0.89	0.0431	Alkaliphilus	-0.8899	0.0431	Flavobacterium	-0.8784	0.05	Blautia	0.8973	0.0389
				Clostridium	0.9402	0.0174							Ruminococcus	0.9419	0.0167
				Aquimarina	0.9332	0.0205							Legionella	0.9301	0.022
				Prevotella	-0.8917	0.0421							Sutterella	-0.8826	0.0474
				Parapedobacter	-0.9127	0.0305									
**Ruminococcus**				Blautia	0.8919	0.042	Mucispirillum	0.9416	0.0168	Flavobacterium	-0.9465	0.0147	Blautia	0.9817	0.003
				Desulfovibrio	0.89	0.0431	Bacteroides	-0.9326	0.0208				Odoribacter	0.8885	0.0439
				Clostridium	0.9725	0.0054							Desulfovibrio	0.9419	0.0167
				Parabacteroide	-0.9797	0.0035							Oscillospira	0.981	0.0031
				Porphyromonas	-0.9535	0.0119							Dehalobacterium	0.968	0.0068
				Sutterella	-0.9387	0.018							Lactobacillus	-0.902	0.0363
													Sutterella	-0.9808	0.0032
**Oscillospira**	Butyricimonas	0.9742	0.005	Flavobacterium	0.9159	0.0289	Clostridium	0.9448	0.0154				Blautia	0.992	0.0009
	Parabacteroides	0.9077	0.0332	Natronincola	0.9156	0.0291							Odoribacter	0.8982	0.0384
	Helicobacter	-0.9503	0.0132										Ruminococcus	0.981	0.0031
	Mucispirillum	-0.9104	0.0317										Pelagicoccus	0.9093	0.0323
	Flexispira	-0.8849	0.0461										Natronincola	0.9088	0.0326
													Dehalobacterium	0.9961	0.0003
													Sutterella	-0.961	0.0092
**Porphyromonas**															
	Bacteroides	0.9259	0.0239	Parabacteoides	0.9595	0.0097	Prevotella	0.9962	0.0003	Lactobacillus	-0.9561	0.011			
	Paraprevotella	0.9147	0.0295	Sutterella	0.9625	0.0087	Paraprevotella	0.9428	0.0163	Clostridium	-0.9668	0.0072			
	Pelagicoccus	-0.9753	0.0046	Odoribacter	-0.9658	0.0076									
				Ruminococcus	-0.9535	0.0119									
				Johnsonella	-0.888	0.0442									

Continued Supplementary Table 6.

**Mucillispirillum**															
	Helicobacter	0.9828	0.0027	Helicobacter	0.9463	0.0148	Ruminococcus	0.9416	0.0168	Flexispira	0.9189	0.0274			
	Flexispira	0.9736	0.0051	Flexispira	0.9505	0.0131	Bacteroides	-0.9872	0.0017	Bacteroides	-0.9928	0.0007			
	Natronincola	0.9756	0.0046	Legionella	0.9753	0.0046				Odoribacter	-0.9251	0.0243			
	Odoribacter	-0.9246	0.0246							Prevotella	-0.9603	0.0094			
	Oscillospira	-0.9104	0.0317							Paraprevotella	-0.9115	0.0312			
**Rikenella**	Dehalobacteriun	-0.9075	0.0333	Anaeroplasma	0.9728	0.0053	Blautia	-0.8876	0.0445	Johnsonella	-0.8983	0.0384	Pelagicoccus	-0.9095	0.0323
													Natronincola	-0.9534	0.012
**Flavobacterium**	Dehalobacteriun	0.8812	0.0483	Oscillospira	0.9159	0.0289	Pelagicoccus	0.9271	0.0233	Blautia	-0.9207	0.0265	Prevotella		
				Natronincola	0.9817	0.003	Johnsonella	-0.8999	0.0374	Desulfovibrio	-0.8784	0.05			
										Ruminococcus	-0.9465	0.0147			
**Sutterella**	Parabacteroides	0.9043	0.035	Parabacteroide	0.9787	0.0037	Olivibacter	0.8849	0.0461				Lactobacillus	0.9664	0.0073
				Olivibacter	0.943	0.0162	Anaeroplasma	0.9623	0.0087				Blautia	-0.947	0.0145
				Porphyromonas	0.9625	0.0087							Desulfovibrio	-0.8826	0.0474
				Odoribacter	-0.9066	0.0338							Oscillospira	-0.961	0.0092
				Ruminococcus	-0.9387	0.018							Ruminococcus	-0.9808	0.0032
													Pelagicoccus	-0.9356	0.0194
													Dehalobacterium	-0.9408	0.0171
**Parapedobacter**	Flexispira	-0.8831	0.0471	Desulfovibrio	-0.9127	0.0305	Blautia	0.899	0.0379						
	Oribacterium	-0.9159	0.0289												
**Flexispira**	Helicobacter	0.9834	0.0026	Helicobacter	0.9969	0.0002	Helicobacter	0.9544	0.0116	Helicobacter	-0.9191	0.0273	Helicobacter	0.9961	0.0003
	Mucispirillum	-0.8849	0.0461	Mucispirillum	0.9505	0.0131	Lactobacillus	0.9185	0.0276	Mucispirillum	0.9189	0.0274	Legionella	-0.9105	0.0317
	Oribacterium	0.9121	0.0308	Legionella	0.9815	0.003	Olivibacter	0.8956	0.0398	Paraprevotella	-0.9352	0.0196			
	Natronincola	0.9586	0.0101	Aquimarina	0.959	0.0099	Aquimarina	0.9539	0.0118	Dehalobacterium	-0.8846	0.0462			
	Odoribacter	-0.8947	0.0404	Bacteroides	-0.9104	0.0318				Prevotella	0.9402	0.0174			
	Oscillospira	0.9736	0.0051												
	Parapedobacter	-0.8831	0.0471												
	Dehalobacteriun	-0.8826	0.0474												
**Pelagicoccus**	Bacteroides	-0.9844	0.0023	Johnsonella	0.9627	0.0086	Alkaliphilus	0.9641	0.0081	Alkaliphilus	0.9902	0.0012	Oscillospira	0.9093	0.0323
	Porphyromonas	-0.9753	0.0046				Flavobacterium	0.9271	0.0233	Anaeroplasma	0.9789	0.0037	Oribacterium	0.9327	0.0207
	Paraprevotella	-0.9611	0.0091							Natronincola	-0.9398	0.0176	Natronincola	0.946	0.015
													Dehalobacterium	0.9	0.0374
													Lactobacillus	-0.9254	0.0242
													Rikenella	-0.9095	0.0323
													Sutterella	-0.9356	0.0194
**Alkaliphilus**				Anaeroplasma	0.9389	0.018	Pelagicoccus	0.9641	0.0081	Anaeroplasma	0.9902	0.0012	Anaeroplasma	0.9259	0.0239
							Desulfovibrio	-0.8899	0.0431	Pelagicoccus	0.993	0.0007			
										Natronincola	-0.9277	0.0231			
**Clostridium**				Desulfovibrio	0.9402	0.0174	Oscillospira	0.9448	0.0154	Porphyromonas	-0.9668	0.0072	Prevotella	-0.8898	0.0432
				Ruminococcus	0.9725	0.0054	Johnsonella	0.9409	0.0171						
				Aquimarina	0.9613	0.0091									
				Parabacteroide	-0.9322	0.021									
				Bacteroides	-0.8836	0.0469									
**Natronincola**	Helicobacter	0.967	0.0072	Oscillospira	0.9156	0.0291	Blautia	0.9124	0.0307	Desulfovibrio	0.8965	0.0393	Oscillospira	0.9088	0.0326
	Mucispirillum	0.9756	0.0046	Flavobacterium	0.9817	0.003	Odoribacter	-0.8864	0.0452	Pelagicoccus	-0.9398	0.0176	Pelagicoccus	0.946	0.015
	Flexispira	0.9586	0.0101							Alkaliphilus	-0.9277	0.0231	Oribacterium	0.9092	0.0324
	Odoribacter	-0.9371	0.0188							Anaeroplasma	-0.9454	0.0152	Dehalobacterium	0.9078	0.0332
													Rikenella	-0.9534	0.012
**Paraprevotella**	Bacteroides	0.9472	0.0144	Blautia	-0.8936	0.041	Prevotella	0.9515	0.0127	Helicobacter	-0.8864	0.0452			
	Porphyromona	0.9147	0.0295				Porphyromonas	0.9428	0.0163	Mucispirillum	-0.9115	0.0312			
	Pelagicoccus	-0.9611	0.0091				Butyrivcimonas	-0.9485	0.0139	Flexispira	-0.9352	0.0196			
**Anaeroplasma**				Rikenella	0.9728	0.0053	Sutterella			Alkaliphilus	0.9789	0.0037	Alkaliphilus	0.9259	0.0239
				Alkaliphilus	0.9389	0.018				Pelagicoccus	0.993	0.0007			
										Natronincola	-0.9454	0.0152			
**Oribacterium**	Helicobacter	0.9145	0.0296				Parabacteroides						Pelagicoccus	0.9327	0.0207
	Flexispira	0.9121	0.0308										Natronincola	0.9092	0.0324
	Bacteroides	-0.8837	0.0468												
	Parapedobacter	-0.9159	0.0289												
**Legionella**				Helicobacter	0.9885	0.0015							Blautia	0.8968	0.0392
				Mucispirillum	0.9753	0.0046							Odoribacter	0.9457	0.0151
				Flexispira	0.9815	0.003							Desulfovibrio	0.9301	0.022
				Aquimarina	0.8906	0.0427							Helicobacter	-0.9241	0.0248
													Flexispira	-0.9105	0.0317
**Butyricimonas**	Parabacteroides	0.9583	0.0102				Paraprevotella	-0.9485	0.0139	Olivibacter	0.9197	0.027			
	Oscillospira	0.9742	0.005												
**Johnsonella**	Dehalobacteriun	0.9104	0.0318	Odoribacter	0.9247	0.0245	Clostridium	0.9409	0.0171	Rikenella	-0.8983	0.0384			
				Pelagicoccus	0.9627	0.0086	Flavobacterium	-0.8999	0.0374						
				Porphyromonas	-0.888	0.0442	Dehalobacterium	-0.9526	0.0123						
**Aquimarina**				Helicobacter	0.944	0.0158	Lactobacillus	0.9095	0.0322	Parabacteroides	0.9407	0.0172			
				Desulfovibrio	0.9332	0.0205	Flexispira	0.9539	0.0118						
				Flexispira	0.959	0.0099									
				Clostridium	0.9613	0.0091									
				Legionella	0.8906	0.0427									
				Bacteroides	-0.9317	0.0212									
**Dehalobacterium**	Flavobacterium	0.8812	0.0483				Johnsonella	-0.9526	0.0123	Flexispira	-0.8846	0.0462	Blautia	0.9947	0.0005
	Johnsonella	0.9104	0.0318										Odoribacter	0.9079	0.0331
	Helicobacter	-0.8897	0.0432										Oscillospira	0.9961	0.0003
	Mucispirillum	-0.9075	0.0333										Ruminococcus	0.968	0.0068
	Flexispira	-0.8826	0.0474										Pelagicoccus	0.9	0.0374
													Natronincola	0.9078	0.0332
													Sutterella	-0.9408	0.0171

Supplementary Table 7. Daily intake of polyphenols for each diet treatment. The intake of polyphenols (expressed in mg *per* day) was calculated considering the amounts of each class of compound assuming the daily food intake of about 3.5 grams *per* mouse.

Daily Intake (mg polyphenols *per* day)

Polyphenols	WT tomato diet (1%)	Res Tom diet (1%)	Indigo diet (1%)	Bronze diet (1%)	White grape skin diet (10%)	Black grape skin diet (10%)
Flavonols	0.014	0.087	0.700	0.525	0.210	0.490
Stilbenes	-	0.315	-	0.195	0.038	0.210
Anthocyanins	-	-	0.175	0.080	-	1.750
Total	0.014	0.402	0.875	0.800	0.248	2.450

Supplementary References

1. Re, R., Pellegrini, N., Proteggente, A., Pannala, A., Yang, M., Rice-Evans, C. (1999). Antioxidant activity applying an improved ABTS radical cation decolorization assay. *Free Radic. Biol. Med.* 26, 1231-1237.

2. Dudonné, S., Vitrac, X., Coutière, P., Woillez, M., Mérillon, J.M. (2009). Comparative study of antioxidant properties and total phenolics content of 30 plant extracts of industrial interest using DPPH, ABTS, FRAP, SOD and ORAC assays. *J. Agric. Food Chem*. 57, 1768-1774.

3. Lenucci, M.S., Cadinu, D., Taurino, M., Piro, G., Dalessandro, G. (2006). Antioxidant composition in cherry and high-pigment tomato cultivars. *J. Agric. Food Chem*. 54, 2606–2613.

4. Lenucci, M.S., Caccioppola, A., Durante, M., Serrone, L., Leonardo, R., Piro, G., Dalessandro, G. (2010). Optimisation of biological and physical parameters for lycopene supercritical CO2 extraction from ordinary and high-pigment tomato cultivars. *J. Sci. Food Agric*. 90, 1709-1718.

5. Garcedo M.C. & Luh B.S. (1986). HPLC analysis of organic acids and sugars in tomato juice. *J. Food Sci*. 51, 571-573.

6. Zhang, J., Kobert, K., Flouri, T. & Stamatakis, A. (2014). PEAR: a fast and accurate Illumina Paired-End read merger. *Bioinformatics* 30, 614-620.

7. Edgar, RC. (2010). Search and clustering orders of magnitude faster than BLAST. *Bioinformatics* 26, 2460-2461.

8. Edgar, RC. (2013). UPARSE: highly accurate OUT sequences from microbial amplicon reads. *Nat. Methods* 10, 996-998.

9. Edgar, RC., Haas, BJ., Clemente, JC., Quince, C.& Knight, R. (2011). UCHIME improves sensitivity and speed of chimera detection. *Bioinformatics* 27, 2194-2200.

10. Andreotti, R., Pérez de León, A.A., Dowd, S.E., Guerrero, F.D., Bendele, K.G., Scoles, G.A. (2011). Assessment of bacterial diversity in the cattle tick Rhipicephalus (Boophilus) microplus through tag-encoded pyrosequencing. *BMC Microbiol.* 11, 6. Doi: 10.1186/1471-2180-11-6.
